# Effect of Risk-Stratified Care on Disability Among Adults With Low Back Pain Treated in the Military Health System: A Randomized Clinical Trial

**DOI:** 10.1001/jamanetworkopen.2023.21929

**Published:** 2023-07-06

**Authors:** Daniel I. Rhon, Tina A. Greenlee, Emily Poehlein, Jason M. Beneciuk, Cynthia L. Green, Ben R. Hando, John D. Childs, Steven Z. George

**Affiliations:** 1 Department of Rehabilitation Medicine, Brooke Army Medical Center, JBSA Fort Sam Houston, Texas; 2 Department of Rehabilitation Medicine, Uniformed Services University, Bethesda, Maryland; 3 Department of Biostatistics and Bioinformatics, Duke University School of Medicine, Durham, North Carolina; 4 Department of Physical Therapy, College of Public Health & Health Professions, University of Florida, Gainesville; 5 Brooks Rehabilitation Clinical Research Center, Jacksonville, Florida; 6 Department of Biostatistics and Bioinformatics and Duke Clinical Research Institute, Duke University School of Medicine, Durham, North Carolina; 7 Department of Orthopaedics and Rehabilitation, Wilford Hall Ambulatory Surgical Center, JBSA Lackland, Texas; 8 Evidence in Motion, San Antonio, Texas; 9 Department of Orthopaedic Surgery and Duke Clinical Research Institute, Duke University, School of Medicine, Durham, North Carolina

## Abstract

**Importance:**

Tailored treatments for low back pain (LBP) based on stratifying risk for poor prognosis have emerged as a promising approach to improve quality of care, but they have not been validated in trials at the level of individual randomization in US health systems.

**Objective:**

To assess the clinical effectiveness of risk-stratified vs usual care on disability at 1 year among patients with LBP.

**Design, Setting, and Participants:**

This parallel-group randomized clinical trial enrolled adults (ages 18-50 years) seeking care for LBP with any duration in primary care clinics within the Military Health System from April 2017 to February 2020. Data analysis was conducted from January to December 2022.

**Interventions:**

Risk-stratified care, in which participants received physiotherapy treatment tailored for their risk category (low, medium, or high), or usual care, in which care was determined by participants’ general practitioners and may have included a referral to physiotherapy.

**Main Outcomes and Measures:**

The primary outcome was the Roland Morris Disability Questionnaire (RMDQ) score at 1 year, with planned secondary outcomes of Patient-Reported Outcomes Measurement Information System (PROMIS) Pain Interference (PI) and Physical Function (PF) scores. Raw downstream health care utilization was also reported within each group.

**Results:**

Analysis included 270 participants (99 [34.1%] female participants; mean [SD] age, 34.1 [8.5] years). Only 21 patients (7.2%) were classified as high risk. Neither group was superior on the RMDQ (least squares [LS] mean ratio of risk-stratified vs usual care: 1.00; 95% CI, 0.80 to 1.26), the PROMIS PI (LS mean difference, −0.75 points; 95% CI −2.61 to 1.11 points), or the PROMIS PF (LS mean difference, 0.05 points; 95% CI, −1.66 to 1.76 points).

**Conclusions and Relevance:**

In this randomized clinical trial, using risk stratification to categorize and provide tailored treatment for patients with LBP did not result in better outcomes at 1 year compared with usual care.

**Trial Registration:**

ClinicalTrials.gov Identifier: NCT03127826

## Key Points

**Question:**

Is risk-stratified care more effective than usual care for patients with low back pain seeking care in the Military Health System?

**Findings:**

In this randomized clinical trial of 270 adults with low back pain, the risk-stratified care approach did not result in superior scores on the Roland Morris Disability Questionnaire at 1 year compared with patients who received usual care.

**Meaning:**

These findings suggest that clinicians seeing patients for low back pain in the Military Health System should be cautious about implementing this particular risk-stratified care treatment approach.

## Teaser

This randomized clinical trial assesses the clinical effectiveness of risk-stratified vs usual care on disability at 1 year among patients with low back pain treated in the Military Health System.

## Introduction

Low back pain (LBP) is among the most frequent causes of medical visits, chronic pain, and lost productivity in the world and, as such, imposes a large societal burden globally.^1,2^ Clinical practice guidelines for LBP endorse a stepped care approach, where ideally care starts with low-risk, low-cost interventions^3^ and then escalates to more invasive treatments.^4,5^ In contrast, risk-stratified care aims to deliver care based on individualized risk for poor prognosis.^6,7^

The Subgroups of Targeted Treatment for Back Pain (STarT Back) tool is the most investigated risk stratification approach for LBP.^8^ The initial trial conducted in United Kingdom found that risk-stratified care reduced disability and decreased health care costs compared with usual care.^8^ Subsequent randomized trials in the United States (ie, the MATCH and TARGET trials)^9,10^ and Denmark^11^ did not find this same benefit.

Thus, whether risk-stratified care is scalable to health systems outside the United Kingdom remains a challenge for improving LBP outcomes. Accordingly, we conducted the Validation of the STarT Back Screening Tool in the Primary Care Management of Low Back Pain in the Military Health System (V-START MHS) clinical trial. It complements existing trials of risk-stratified care by being, to our knowledge, the first in the United States to use individual randomization and to occur in a single-payer health system. The primary aim was to assess the clinical effectiveness of risk-stratified vs usual care in patients with LBP. We hypothesized larger improvements in disability for patients randomized to receive risk-stratified care. A secondary aim was to report 12-month health care utilization in each treatment group.

## Methods

### Trial Design

This was a parallel-group randomized clinical trial with participants randomized 1:1 to receive risk-stratified or usual care. Ethics approval was provided by the institutional review board at Brooke Army Medical Center. The trial was reported using the Consolidated Standards of Reporting Trials (CONSORT) checklist, the statistical analysis plan was published a priori,^12^ and the study protocol is available in Supplement 1. Written informed consent was obtained from all participants.

### Setting and Participants

Participants were patients seeking care for LBP in 2 large hospitals in the US MHS in Texas, enrolled between April 2017 and February 2020. The MHS is a single-payer government health system and one of the largest health systems in the United States. Participants were adults aged 18 to 50 years with a primary concern of LBP for any duration (with or without radiculopathy). Individuals were excluded if they had history of spine surgery in the last 24 months, received any prior care for this episode of LBP (within last 6 months), pending litigation, pregnancy within last 6 months, or LBP symptoms associated with potentially serious systemic disorders or illness (eg, malignant neoplasm, infection, inflammatory arthritis, cauda equina syndrome).

### Randomization

Participants were randomized 1:1 into either usual care or risk-stratified care via sequentially numbered opaque sealed envelopes. The randomization sequence was determined with an electronic random number generator developed by a statistician not participating in the study. Randomization was performed in permuted blocks of 2 or 4 with random variation of the blocking number. Research coordinators provided the randomization number to the research assistants after signed consent and enrollment of eligible participants. Data were collected in person at baseline and by electronic survey for follow-up visits. Investigators did not see the data until study completion. Clinicians delivering the treatment and participants could not be blinded.

### Allocation

Treatment allocation was tracked and coded locally at each site, and the number of patients in each group was balanced for each site. Statisticians responsible for primary and secondary analyses were blinded to treatment assignment until the statistical analysis plan was completed and signed, analyses completed, and tables produced with primary outcome results.

### Intervention

Full details of the risk-stratified intervention are reported according to the Template for Intervention Description and Replication checklist^13^ in Table 1, and a description of the psychologically informed physiotherapy training has been published elsewhere.^14,15,16,17,18^ In short, patients were categorized as STarT Back Tool low-, medium-, or high-risk groups and received a physiotherapy treatment plan tailored for each risk category. For patients receiving usual care, nothing was done to alter regular care determined by their general practitioner, which could have also included a referral to physiotherapy. If patients in the usual care group were referred to physiotherapy, they received care from a physiotherapist not trained or participating in provision of care for patients in the risk-stratified care group. Further details of the usual care intervention are reported in eTable 10 in Supplement 2.^19^

**Table 1.** Template for Intervention Description and Replication Recommended Reporting Elements Describing Intervention of the Trial^a^

Item No. and name	Description
1. Brief name	Risk-stratified care for low back pain (based on low-, medium-, and high-risk categories of the STarT Back screening tool)
2. Why	Prior work suggests psychosocial profile at baseline is an indicator of prognosis. Instead of trying to determine a specific diagnosis and targeting treatment to the diagnosis, this approach instead focuses on the prognosis of the patient regardless of what the specific patho-mechanical diagnosis might be. Treatment is then tailored to 1 of 3 different risk categories. Patients at lower risk for poor prognosis usually need less intervention, and patients at higher risk often need more intervention. It is an approach designed to make more efficient use of health care resources by targeting the intensity of care based on perceived need.
3. What materials	Patients fill out a 9-item questionnaire. The score places the individual in 1 of 3 treatment categories based on risk for poor prognosis (low, medium, or high).
4. What procedures	All groups Evidence-based assessment of low back pain according to current clinical practice guidelines, including the limited use of imaging except for patients with red flags Enhanced active management advice emphasizing positive messages about activity, pain relief, and work for lower back pain Reassurance to address specific concerns related to their lower back pain and implications on work A copy of the *Back Book* and view a 5-minute video based on the *Back Book* entitled “The Truth About LBP”^b^ Low-risk group The aforementioned items with a 2-item spinal manipulation screening, with spinal manipulation delivered in primary care if indicated^c^ No referral for ongoing physical therapy Medium-risk group Same as low-risk group with referral for ongoing physical therapy based on the Treatment Based Classification principles for up to 8 visits, 30-60–minute sessions (twice weekly)^d^ High-risk group Same as low- and medium-risk groups, and patient is referred for ongoing physical therapy using Treatment Based Classification principles for up to 12 visits, 45-60–minute sessions (twice weekly)^d^ Physical therapy is psychologically augmented with the assessment of biopsychosocial risk factors and the adoption of cognitive behavioral principles that explore patient concerns and address unhelpful beliefs and behaviors. These strategies include tailored education, graded exercise, graded exposure, among others. Details of the content of the psychologically informed physical therapy are available elsewhere^14^ and have been used in other trials.^9^
5. Who provided	Primary care clinicians included physicians, nurse practitioners, and physicians’ assistants. The primary care clinician would initially see the patient, provide education, and refer those they deemed eligible to participate in the study. Once in the study, to ensure a minimum baseline of education for all participants, everyone received the education described in “All groups” from item 4. Licensed and credentialed physical therapists then provided the additional care for all 3 risk groups. These individuals included a variety of backgrounds, years of practice, and expertise levels. The physical therapists delivering the risk-stratified care attended a 2-d course that provided training for the delivery of care to patients in this treatment group (psychologically informed physical therapy, vignettes, case studies, practical application).
6. How	The physical therapists received the risk stratification score from the research team and delivered care in the clinic.
7. Where	Care took place in the primary care/family medicine clinic or physical therapy clinic. Treatment for low-risk care often took place in the primary care clinic, and care for medium- and high-risk groups took place in the outpatient physical therapy clinics.
8. When and how much	The specific number of sessions was not controlled or directed in this study. The risk-stratified care is designed to be minimal visits for low risk (1-2), more visits for medium risk (6-8) and the most visits for the high-risk group (10-12); however, this was left up to the individual physical therapist to determine as they felt most appropriate based on the individual information they had about the patient and training they had received. The longest window of care should have been approximately 6 weeks, with the goal of at least twice a week for patients in medium- and high-risk groups. Actual number of visits in each group is outlined in eTables 2 and 3 in Supplement 2.
9. Tailoring	The treatment was tailored according to the risk category of each individual patient (low, medium, or high) as described previously. Within the same risk category, the amount of care was not controlled within the study and left up to the clinician to determine, keeping in consideration all that they had learned in the training about the needs of patients within each of the 3 risk categories.
10. Modifications	The treatment plan was not modified, but the treatment likely varied somewhat from patient to patient and between clinicians, as they had the freedom to deliver care as they thought best. The study manipulated the training received by the participating physical therapists, asking them to treat their patients within the tenants of risk-stratified care.
11. How well (planned)	There was no plan to assess intervention adherence or fidelity a priori, as the number and exact content of sessions was expected to vary across patients and even some within the same risk category.
12. How well (actual)	Treatment fidelity was tracked at the end of the study using data from electronic medical records stored in the Military Health System Data Repository. This allowed for the calculation of the number of back pain–related physical therapy visits for every participant but not the specific content of each visit. The total number of physical therapy visits could then be a proxy for treatment fidelity (fewest visits for individuals in the low-risk group; most visits for individuals in the high-risk group).

Abbreviation: STarT Back, Subgroups of Targeted Treatment for Back Pain.

^a^ This table provides details for the risk-stratified care based on guidance from the Template for Intervention Description and Replication checklist. Details of usual care are listed in eTable 6 in Supplement 2 according to current recommendations for reporting and describing usual care treatment groups.

^b^ Klaber Moffett et al,^15^ 2002; Clinically Relevant Technologies, 2018.^16^

^c^ Spinal manipulation described in Fritz et al,^17^ 2005.

^d^ Treatment Based Classification described in Delitto et al,^18^ 2012.

### Measures

#### STarT Back Screening Tool

The 9-item STarT Back Screening Tool (SBST) was used to stratify patients based on risk (low, medium, high) for persistent disabling symptoms.^8^ The type and intensity of treatment was then tailored based on SBST risk category for those randomized to risk-stratified care.

#### Demographic Characteristics, Medical History, and Care Expectations

Patients self-reported demographic characteristics, including age, sex, race (Asian, Black or African American, Native Hawaiian or Pacific Islander, White, ≥1 race, and other [self-reported or uses the label other assigned within the Defense Enrollment and Eligibility System]) and ethnicity (Hispanic or Latino and not Hispanic of Latino), job title, employment history, and current employment status. The Credibility/Expectancy Questionnaire (CEQ) evaluating treatment credibility and expectations for improvement was also assessed at baseline.^20^

#### Fidelity of Risk-Stratified Care

We assessed treatment fidelity by number of LBP-related visits within the first 90 days (eTable 3 in Supplement 2). We expected low-risk patients to have the fewest and high-risk patients to have the highest number of visits, consistent with the risk category.^14,21^ We also assessed psychological distress as an indirect indicator of treatment fidelity (eTable 4 in Supplement 2). Psychological distress can mediate outcomes and is expected to decrease over time when properly addressed.^22,23^ Therefore we monitored levels of distress with the Optimal Screening for Prediction of Referral and Outcomes Yellow Flag (OSPRO-YF) tool^24^ as a proxy measure for whether treatment was being delivered as intended.

#### Outcomes

The primary outcome was the Roland Morris Disability Questionnaire (RMDQ) at 12 months. The RMDQ is reliable, valid, and responsive to change for patients with both acute and chronic LBP^25,26,27^ and was chosen because it was also used in the original STarT Back trial^8^ allowing direct comparison of results.

Secondary outcomes included the Patient-Reported Outcomes Measurement Information Systems (PROMIS)–57 profile, a collection of short forms capturing 7 important health domains. PROMIS measures are reliable and valid for measuring change in patients with LBP.^28,29^ The measures generate T scores, representing standard scores with a mean of 50 and standard deviation of 10 in a reference population. The Physical Function and Pain Interference domains were used as secondary outcomes. The Sleep Disturbance domain was also collected due to its recommendation as a core outcome in pain trials but was not formally analyzed (eTable 8 in Supplement 2).^30,31^ The RMDQ, PROMIS, and OSPRO scores were assessed at baseline, 6 weeks, 6 months, and 12 months. We also collected health care utilization events extracted from the MHS Data Repository (MDR). The MDR captures data from electronic medical records and claims data for all outpatient encounters at the single-person level where TRICARE is the payer. This allowed capture of care even when patients moved or sought care from other locations. It includes all back-pain related consultations and procedures documented in electronic medical records with relevant *Current Procedural Terminology*, Healthcare Common Procedure Coding System, and *International Statistical Classification of Diseases and Related Health Problems, Tenth Revision (ICD-10)* codes as well as all medication prescriptions. We also calculated total LBP-related visits and costs as well as total medical costs.

### Sample Size

The sample size calculation was based on the ability to detect a between-group effect size of 0.3 at 12 months on the RMDQ. Based on a 2-tailed significance level at an α level equal to .05, 80% power, and allowing for a 25% loss to follow-up (not uncommon in military settings with high deployment rates), we aimed to recruit 290 participants, 145 in each group.

### Statistical Analysis

#### Main Analysis

Continuous data are presented using the mean and SD or median with IQR based on data distribution, while categorical data are presented using count (percentage) of nonmissing data. The primary statistical analysis plan was described according to current reporting guidelines^32^ and published openly^12^ before any raw data was delivered to the team of statisticians. Treatment allocation was blinded in the data provided to the statisticians, with the designation only of Treatment A and Treatment B given to each intervention group. First, RMDQ at each time point was compared between the 2 treatment groups in the safety population (everyone who received at least some treatment). Then, using the modified intention-to-treat (mITT) population (patients with a baseline score and at least 1 follow-up), treatment effect estimates were obtained using a repeated measures model for RMDQ score at each point including treatment, time (categorical), and an interaction between treatment and time with adjustment for baseline score as a covariate using an unstructured covariance matrix and degrees of freedom by Kenward-Rodger. A negative binomial probability distribution with a log link function was chosen based on skewed data identified using residuals and other graphical methods. As a result, geometric mean ratios were used as a measure of treatment difference. Least-squares means (LS means) for each treatment group and the LS mean ratio between the 2 are reported with 95% CIs and 2-sided *P* values. PROMIS Pain Interference and Physical Function scores were similarly analyzed using a normal probability distribution with identity link function and reported using LS means for each treatment group and the LS mean difference with 95% CIs. Health care utilization was summarized and presented by group for the number of visits and risk ratios for the other binary outcomes. Observed unadjusted cost values were also summarized. Statistical analyses were conducted using SAS version 9.4 (SAS Institute), and *P* < .05 was considered statistically significant.

#### Sensitivity Analyses

We conducted a series of sensitivity analyses to test various assumptions. First, we ran the primary model with additional adjustment for age, sex, and active-duty status, as these were potential confounders identified a priori. Second, we ran the primary model excluding everyone without an RMDQ score at 1 year (25 of 270 [9.3%]) and then excluding individuals who reported their ability to access medical care had been affected by the COVID-19 pandemic (38 [14.1%]). Finally, we conducted a subgroup analysis comparing the primary outcome according to SBST risk group.

## Results

Study recruitment, enrollment, randomization, and follow-up are reported in the Figure. Of the 290 patients randomized, 270 had at least 1 postbaseline RMDQ score and were included in the primary analysis (134 in usual care; 136 in risk-stratified care). The mean (SD) age of participants was 34.1 (8.5) years, 99 (34.1%) were female participants, and 243 (84.1%) were on active military duty status. For SBST risk status, there were no obvious postrandomization imbalances noted; most patients were low risk (160 [55.2%]), with only 21 (7.2%) in the high-risk category. Remaining demographic and baseline descriptive variables are presented in Table 2. Mean CEQ scores were similar in both groups and therefore not used as covariates in any analyses. Baseline characteristics of those with missing RMDQ were similar to those that did not have missing scores (eTable 1 in Supplement 2).

**Figure.**
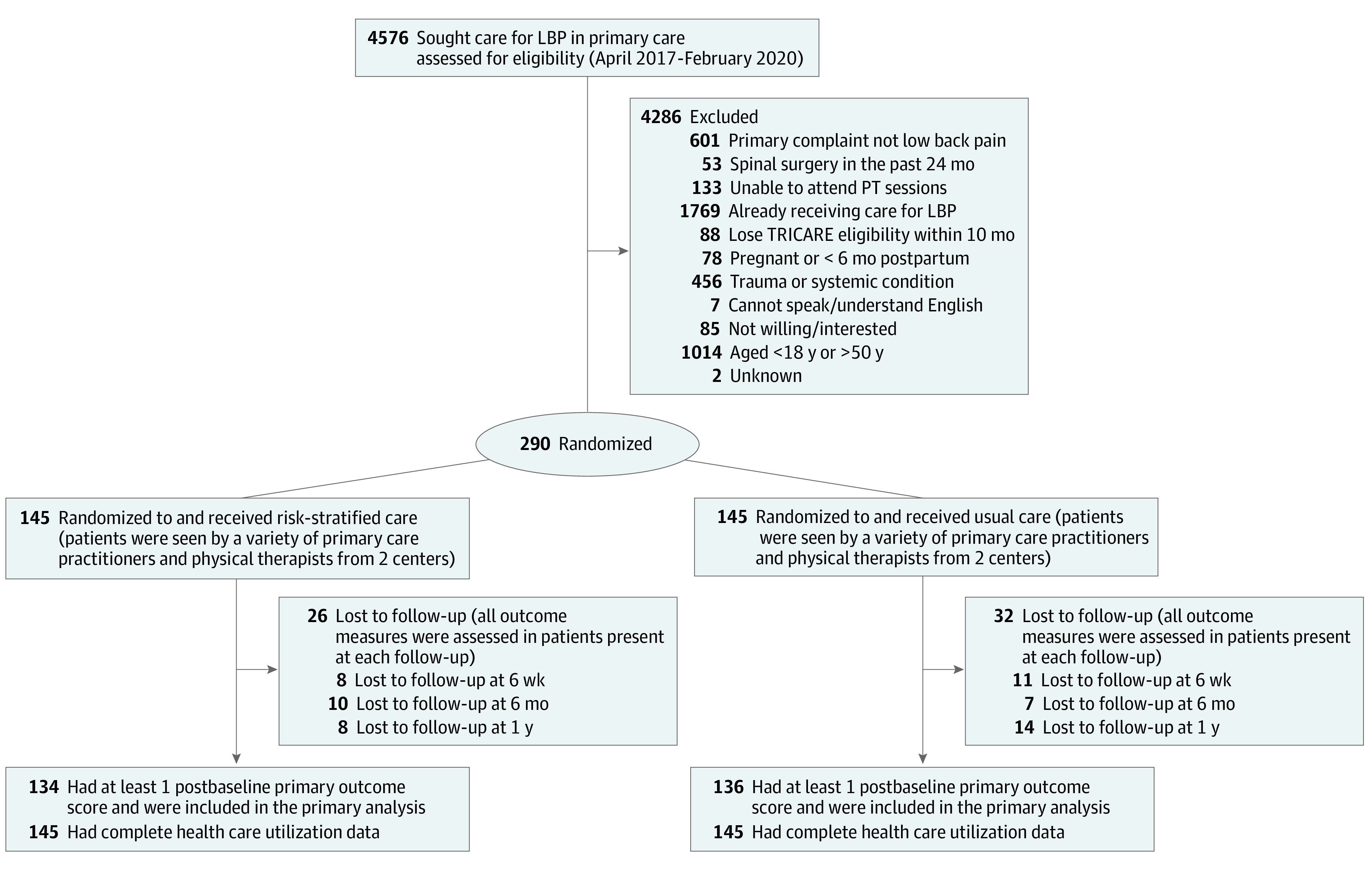
Study Flow Diagram Lost to follow-up values represent the total number of participants lost to follow-up at each time point in each group and are not summative values. Some participants may have missed an early follow-up but still kept a later one. LBP indicates lower back pain; PT, physiotherapy.

**Table 2.** Baseline Characteristics by Treatment Group

Characteristic	Patients, No. (%)		
	Usual care (n = 145)	Risk-stratified care (n = 145)	Total (n = 290)
Age, mean (SD), y	33.6 (8.8)	34.7 (8.1)	34.1 (8.5)
Sex			
Female	48 (33.1)	51 (35.2)	99 (34.1)
Male	97 (66.9)	94 (64.8)	191 (65.9)
Race			
Asian	10 (6.9)	8 (5.5)	18 (6.2)
Black or African American	30 (20.7)	28 (19.3)	58 (20.0)
American Indian, Alaska Native, Native Hawaiian, or Pacific Islander	1 (0.7)	4 (2.8)	5 (1.7)
White	80 (55.2)	77 (53.1)	157 (54.1)
≥1 Race	11 (7.6)	11 (7.6)	22 (7.6)
Other^a^	13 (9.0)	17 (11.7)	30 (10.3)
Hispanic/Latino ethnicity	42 (29.0)	44 (30.3)	86 (29.7)
Education level			
GED	3 (2.1)	0	3 (1.0)
High school	48 (33.1)	51 (35.2)	99 (34.1)
Associate’s degree	35 (24.1)	34 (23.4)	69 (23.8)
Bachelor’s degree	34 (23.4)	33 (22.8)	67 (23.1)
Master’s degree	17 (11.7)	25 (17.2)	42 (14.5)
Doctorate degree	8 (5.5)	2 (1.4)	10 (3.4)
Employment status			
Full time	138 (95.2)	134 (92.4)	272 (93.8)
Part time	2 (1.4)	3 (2.1)	5 (1.7)
Not employed	5 (3.4)	8 (5.5)	13 (4.5)
Marital status			
Single	28 (19.3)	28 (19.3)	56 (19.3)
Married or living with significant other	105 (72.4)	100 (70.0)	205 (70.7)
Divorced, separated, or widowed	12 (8.3)	17 (11.7)	29 (10.0)
Time in service, mean (SD), mo^b^	143.8 (88.2)	149.3 (88.5)	146.6 (88.2)
Active-duty service member	118 (81.9)	125 (86.2)	243 (84.1)
Tobacco use			
None	126 (86.9)	129 (89.0)	255 (87.9)
Cigarettes	7 (4.8)	4 (2.8)	11 (3.8)
Cigar	1 (0.7)	1 (0.7)	2 (0.7)
Vape	6 (4.1)	3 (2.1)	9 (3.1)
Smokeless or dip	5 (3.4)	7 (4.8)	12 (4.1)
Other	0 (0.0)	1 (0.7)	1 (0.3)
Self-reported physical activity, aerobic days			
<1/wk	18 (12.4)	16 (11.0)	34 (11.7)
1-3/wk	67 (46.2)	55 (37.9)	122 (42.1)
>3/wk	60 (41.4)	74 (51.0)	134 (46.2)
Average physical activity^c^			
1	5 (3.4)	5 (3.4)	10 (3.4)
2	14 (9.7)	15 (10.3)	29 (10.0)
3	54 (37.2)	39 (26.9)	93 (32.1)
4	44 (30.3)	51 (35.2)	95 (32.8)
5	28 (19.3)	35 (24.1)	63 (21.7)
By the end of your treatment, how much improvement in your limitations due to back pain do you really feel will occur (range, 0%-100%)			
Mean (SD)	63.9 (27.8)	69.6 (24.3)	66.8 (26.2)
Median (IQR)	70.0 (50.0-90.0)	80.0 (50.0-90.0)	70.0 (50.0-90.0)
Baseline Credibility Score (range, 3-27), mean (SD)	20.8 (4.7)	21.3 (4.5)	21.1 (4.6)
Baseline Expectancy Score (range, 3-27), mean (SD)	19.1 (5.5)	20.1 (5.0)	19.6 (5.3)
Baseline STarT Back Screening Tool Risk Category			
Low	77 (53.1)	83 (57.2)	160 (55.2)
Medium	57 (39.3)	52 (35.9)	109 (37.6)
High	11 (7.6)	10 (6.9)	21 (7.2)
No. of LBP episodes in the last 5 y^d^			
Mean (SD)	26.1 (149.1)	12.0 (29.5)	19.1 (107.5)
Median (IQR)	3 (1-10)	3 (1-8)	3 (1-10)
Baseline RMDQ score			
Mean (SD)	10.2 (5.8)	9.3 (5.9)	9.8 (5.8)
Median (IQR)	11 (6-14)	8 (5-14)	9 (5-14)

Abbreviations: LBP, low back pain; RMDQ, Roland Morris Disability Questionnaire; STarT Back, Subgroups of Targeted Treatment for Back Pain.

^a^ Other race is either self-reported as such or uses the label other assigned within the Defense Enrollment and Eligibility System.

^b^ This refers to the value for the sponsor, who is the active-duty service member (ie, if participant is a dependent, value refers to the service member sponsor).

^c^ Higher score indicates more activity.

^d^ LBP episode was defined based on answers to this question: “An episode of low back pain could easily last only 1 day or several weeks. A specific episode is over when the symptoms resolve for a period of time. Considering this, how many episodes of back pain have you had in the last 5 years, NOT including this CURRENT EPISODE you are seeking care for?”

### Primary Outcome

eFigures 1 and 2 and eTable 5 in Supplement 2 summarize the primary outcome over time among all participants. Loss to follow-up over time was observed, with 113 of 145 and 119 of 145 participants having RMDQ data available at the final 1-year point in usual and risk-stratified care groups, respectively. Overall, RMDQ scores were similar between groups at each time point. Median (IQR) scores are reported for RMDQ due to skewed distribution.

The RMDQ total score at 12 months for each treatment group as well as the ratio between the 2 treatment groups were estimated (Table 3). There was no significant difference in the RMDQ scores between treatment groups at 1-year (LS mean ratio, 1.00; 95% CI, 0.80-1.26). No differences in treatment effect were observed in any of the sensitivity analyses (Table 3).

**Table 3.** Treatment Differences for Primary and Secondary Outcomes

Outcome	Least-square mean (95% CI)		Treatment effect, least-square mean ratio (95% CI)	*P* value
	Usual care (n = 134)	Risk stratified care (n = 136)		
Primary outcome, RMDQ score at 12 mo	5.16 (4.37 to 6.09)	5.14 (4.36 to 6.07)	1.00 (0.80 to 1.26)	.98
RMDQ score at 12 mo, adjusted for age, sex, and active-duty status^a^	5.36 (4.13 to 6.97)	5.31 (4.10 to 6.88)	1.01 (0.80 to 1.27)	.93
RMDQ score at 12 mo, removing all individuals without 12-mo score^a^	5.12 (4.32 to 6.07)	5.10 (4.30 to 6.04)	1.00 (0.79 to 1.27)	.97
RMDQ score at 12 mo by baseline risk strata^a^^,^^b^				
Low risk (n = 151)	5.68 (3.90 to 8.30)	5.00 (3.53 to 7.09)	1.14 (0.68 to 1.90)	.62
Medium risk (n = 120)	6.68 (5.06 to 8.83)	5.69 (4.09 to 7.91)	1.18 (0.76 to 1.81)	.46
High risk (n = 17)	7.56 (3.54 to 16.13)	12.27 (7.54 to 19.97)	0.62 (0.25 to 1.51)	.27
RMDQ score at 12 mo, removing individuals (n = 25) affected by COVID-19 pandemic^a^	4.74 (3.80 to 5.92)	4.81 (3.83 to 6.04)	0.99 (0.72 to 1.35)	.93
RMDQ score change from baseline at 12 mo	3.87 (2.85 to 4.89)	4.05 (3.05 to 5.05)	−0.18 (−1.61 to 1.25)^c^	.81
Secondary outcomes				
Mean PROMIS Pain Interference at 12 mo	51.74 (50.41 to 58.74)	52.50 (51.21 to 57.95)	−0.75 (−2.61 to 1.11)^c^	.43
Mean PROMIS Physical Function at 12 mo	46.34 (45.11 to 47.58)	46.29 (45.10 to 47.48)	0.05 (−1.66 to 1.76)^c^	.95

Abbreviations: PROMIS, Patient-Reported Outcomes Measurement Information System; RMDQ, Roland Morris Disability Questionnaire.

^a^ Sensitivity or subgroup analyses.

^b^ These results should be interpreted with caution, as the study was not designed or powered to assess differences between risk groups.

^c^ Mean difference of least squares means.

### Secondary Outcomes

PROMIS Physical Function and Pain Interference improved from baseline to 1-year with no significant differences between groups at any time point (eTables 6 and 7 and eFigures 3 and 4 in Supplement 2). Mean differences between treatment groups at 1-year were not significant for PROMIS Physical Function (LS mean difference, 0.05 points; 95% CI, −1.66 to 1.76 points) or PROMIS Pain Interference (LS mean difference, 0.75 points; 95% CI, −2.61 to 1.11) (Table 3).

### Fidelity of Risk Stratification

The mean (SD) number of total medical visits of any kind related to LBP increased by risk strata from 5.8 (6.7), 8.5 (9.9), to 14.6 (9.4) visits in the low-, medium-, and high-risk groups, respectively (eTable 2 in Supplement 1). Trends were the same for care within 90 days (Table 4). Further inspection suggests that the dosing of risk-stratified care was delivered as intended; both the number of total medical visits of any kind for LBP and specifically physiotherapy-related visits were similar for the medium- and high-risk groups, both had more physiotherapy visits than the low-risk group, and the amount of treatment received by the usual care group resembled that received by the low-risk SBST group (eTables 2 and 3 in Supplement 2). However, it is possible that care for medium-risk and high-risk groups was underdosed, and a greater number of visits, aligning with the original treatment plan (approximately 8 visits for medium-risk and approximately 12 visits for high-risk) would have shown a treatment effect. Similarly, the risk-stratified care group had lower psychologic distress at 6 weeks compared with the usual care group (eTable 4 in Supplement 2). OSPRO-YF scores were 32% higher in the usual care group compared with the risk-stratified group (LS mean ratio, 1.32; 95% CI, 1.11-1.56).

**Table 4.** Summary of Health Care Utilization and Costs

Care utilization event (during 1-y follow-up unless otherwise noted)^a^	Participants, No. (%)		
	Usual care (n = 145)	Risk stratified care (n = 145)	Total (n = 290)
Lumbar radiograph			
Any	39 (26.9)	37 (25.5)	76 (26.2)
Within 90 d	26 (17.9)	25 (17.2)	51 (17.6)
Days to first, median (IQR)	21 (0-121)	22 (0-113)	21 (0-118)
Lumbar advanced imaging (MRI or CT)^b^			
Any	30 (20.7)	21 (14.5)	51 (17.6)
Within 90 d	17 (11.7)	5 (3.4)	22 (7.6)
Days to first, median (IQR)	65 (32-190)	143 (82.5-273)	131 (39-241)
Lumbar injection, therapeutic or diagnostic, joint or epidural			
Any	8 (5.5)	6 (4.1)	14 (4.8)
Within 90 d	3 (2.1)	2 (1.4)	5 (1.7)
Days to first, median (IQR)	129 (56-202.5)	171 (72-266)	138 (64-252)
Intramuscular injection, NSAID or muscle relaxer	26 (17.9)	19 (13.1)	45 (15.5)
Within 90 d	12 (8.3)	10 (6.9)	22 (7.6)
Days to first, median (IQR)	82 (0-169)	144 (5.5-301)	90 (0-198)
Opioids			
Filled a prescription	29 (20.0)	32 (22.1)	61 (21.0)
Filled prescription within 90 d	16 (11.0)	18 (12.4)	34 11.7)
Unique fills, median (IQR)^c^	1 (1-3)	2 (1-2)	2 (1-2.5)
Total days’ supply, median (IQR)^c^	6 (4-23.5)	7 (4.5-10)	7 (4-18)
Days to first fill, median (IQR)^c^	83 (2.5-198.5)	84 (21.5-179)	84 (18.5-198.5)
Analgesic lidocaine patches			
Any	33 (22.8)	46 (31.7)	79 (27.2)
Within 90 d	20 (13.8)	29 (20.0)	49 (16.9)
Days to first, median (IQR)	48 (0-182)	20 (0-149)	41 (0-161)
Oral muscle relaxants			
Any	71 (49.0)	71 (49.0)	142 (49.0)
Within 90 d	44 (30.3)	54 (37.2)	98 (33.8)
Days to first, median (IQR)	11 (0-213)	0 (0-90)	3.5 (0-131)
Oral analgesics, NSAIDs and acetaminophen			
Any	117 (80.7)	110 (75.9)	237 (81.7)
Within 90 d	72 (49.7)	74 (51.0)	146 (50.3)
Days to first, median (IQR)	48 (0-171)	27.5 (0-137)	42 (0-146)
LBP-related visits			
Mean (SD)	7.12 (8.76)	7.74 (8.33)	7.34 (8.54)
Median (IQR)	3 (1-11)	5 (2-10)	4 (2-10)
Within 90 d, median (IQR)	1 (0-1)	1 (1-3)	1 (1-2)
LBP-related costs, US $			
Mean (SD)	1463 (2283)	1415 (1581)	1439 (1961)
Median (IQR)	753 (298-1473)	922 (449-1711)	850 (319-1626)
Total medical costs, US $			
Mean (SD)	6951 (10 140)	8403 (11 535)	7677 (10 866)
Median (IQR)	3423 (1446-8551)	4042 (2178-8252)	3735 (1820-8378)

Abbreviations: CT, computed tomography; MRI, magnetic resonance imaging; NSAID, nonsteroidal anti-inflammatory drug; LBP, low back pain.

^a^ All health care utilization variables come from the electronic medical records and claims data in the Military Health System data repository.

^b^ Only 2 individuals in each group (4 total) received a CT scan.

^c^ From only those who received a prescription. No participants in either group underwent a lumbar surgical procedure.

### Health Care Utilization

Table 4 summarizes health care utilization by treatment groups. Rates of utilization events did not differ significantly between groups for radiographs or advanced imaging, and none of the participants received surgical procedures. The median (IQR) number of LBP-related visits during the initial 90-day period after enrollment was 4 (2-10), similar between the 2 treatment groups (Table 4; eTables 2 and 3 in Supplement 2). Individuals in risk-stratified care had higher observed median (IQR) costs compared with those in usual care ($922.20 [$449.30-$1710.70] vs $752.60 [$298.40-$1473.10]). Additionally, the total median (IQR) medical costs were higher among those receiving risk-stratified care ($4041.70 [$2177.70-$8252.20] vs $3423.20 [$1446.3-$8551.10]).

### Adverse Events

No adverse events were reported, serious or otherwise. This was further verified through assessment of the MDR database for other significant care events. Two participants were seen for ureterolithiasis, both in the risk-stratified care group, but these occurred at 278 and 312 days, respectively, after enrollment. Emergency care was sought by 59 in the usual care group and 73 in the risk-stratified care group. However, these visits were related to LBP concerns for only 10 individuals (6.9%) in the usual care group and 7 (4.8%) in the risk-stratified care group. Some participants near the latter part of the trial may have been affected by COVID-19 pandemic. We collected a predeveloped survey^32^ on the impact of COVID-19. Of 39 individuals who answered the survey at 1 year (most completed the study prior to the pandemic), 21 reported at least some impact on their ability to receive health care (eTable 9 in Supplement 2).

## Discussion

Recent calls to action for lessening the societal burden of LBP have prioritized identification of effective treatment approaches that can be scaled across different health systems.^10,14^ Risk-stratified care for LBP using the SBST is one such approach with efficacy demonstrated in 1 health system that has not been reproduced in other settings. Results from this trial in a US single-payer system suggested no benefit for self-reported LBP disability of risk-stratified care over usual care. Furthermore, downstream health care utilization appeared to be no different. These findings suggest that risk-stratified care offers no obvious benefit when implemented in primary care within the MHS.

Risk-stratified care for LBP using the SBST has been tested now in 5 large, randomized trials with mixed results.^8,9,10,11^ Two of these trials randomized at the clinic level^10,14^ and 3 at the individual person level (including the current trial).^33^ Only the original trial demonstrated an advantage of risk-stratified care over usual care.^34^ The V-START MHS trial is an important addition to existing trials, as it was the first, to our knowledge, conducted in the United States within a single-payer system using individual-level randomization. Prior trials in the United States used group randomization and initiated treatment via manual referral from primary care. These designs resulted in many instances where referrals were not placed for risk stratification.^33^ In contrast, the V-START MHS trial avoided this limitation by automating primary care referrals to the study-trained physical therapist; thus, a strength of this trial compared with the others conducted in the United States was enhanced fidelity of risk-stratified care. One key difference for V-START MHS compared with the original STarT Back trial is the age of participants. The mean (SD) age in the original trial was 50.1 (15.0) years compared with 34.1 (8.5) years in the V-START MHS.

Interestingly, there were higher raw costs associated with the risk-stratified approach compared with usual care. This unexpected finding was likely due to the increased amount of physical therapy utilization in the risk-stratified care group because we did not observe differences in the treatment groups for other health care utilization variables, like imaging or surgery. Most patients in the usual care group ended up with referrals to physical therapy outside the framework of the study, but the type and dosing of care they received is unknown. A formal cost-effectiveness analysis may help validate these findings.

### Limitations

This study has limitations. The primary limitation is the low number of patients categorized as high risk. Even though our analyses indicated no differences in treatment effect by risk status, there may not have been enough individuals in the high-risk group (21 [7.2%]) to confidently make this determination. The original trial in the United Kingdom had a 4-fold higher rate of high-risk patients (28%).^8^ Another limitation is that most patients in the usual care group (88 [60.7%]) received physiotherapy outside the framework of the study (eTables 2 and 3 in Supplement 2), although this was almost identical to the 58.0% of the usual care group in the original trial that also received follow-on physiotherapy.^8^ While patients in the usual care group were purposefully scheduled with physical therapists who had not received any of the risk stratification training and not provided the patient’s risk category, there is always potential for treatment contamination as patients were seen in the same clinics. Additionally, because this trial was completed in the MHS, these results may not be generalizable to other health systems. The fidelity of treatment content likely varied, and how well it aligned with psychologically informed physiotherapy principles could not be determined.

## Conclusions

The V-START MHS trial results did not indicate superior outcomes for risk-stratified care compared with usual care for self-reported LBP disability or health care utilization. These findings suggest that clinicians should be cautious about implementing this particular risk-stratified care treatment approach.

## Notes

Supplement 1.
Trial Protocol

Supplement 2. **eFigure 1.** Roland Morris Disability Questionnaire (RMDQ): Between Groups Over Time (LS Means) **eFigure 2.** RMDQ Between-Group Comparison (Observed Values, Raw) **eFigure 3.** PROMIS Pain Interference Between-Groups Over Time (LS Means) **eFigure 4.** PROMIS Physical Function Between-Groups Over Time (LS Means) **eTable 1.** Demographic Differences for Individuals With Only Baseline Data vs Individuals With at Least 1 Follow-up ( mITT Population) **eTable 2.** Number of Low Back Pain (LBP)–Related Visits and Costs By Risk Strata **eTable 3.** Number of Low Back Pain (LBP)–Related Visits by Risk Strata and Treatment **eTable 4.** Summary of OSPRO-Yellow Flag Count by Treatment Group (Observed Values) **eTable 5.** Mean RMDQ Scores at Each Time Point by Treatment Group (Observed Values) **eTable 6.** Mean PROMIS Pain Interference Scores at Each Time Point by Treatment Group (Observed Values) **eTable 7.** Mean PROMIS Physical Function Scores at Each Time Point by Treatment Group (Observed Values) **eTable 8.** Mean PROMIS Sleep Disturbance Scores at Each Time Point by Treatment Group (Observed Values) **eTable 9.** COVID-19 Impact by Treatment Group **eTable 10.** Usual Care Intervention Recommended Reporting Elements
eReferences.

Supplement 3.
Data Sharing Statement
