# Supplementary material for: Effect of Risk-Stratified Care on Disability Among Adults With Low Back Pain Treated in the Military Health System: A Randomized Clinical Trial
Source: JAMA Netw Open. 2023 Jul 6;6(7):e2321929. doi: 10.1001/jamanetworkopen.2023.21929 (PMC10326636; doi:10.1001/jamanetworkopen.2023.21929)
Supplement: Supplement 1. — Trial Protocol [file jamanetwopen-e2321929-s001.pdf]

Brooke Army Medical Center
Institutional Review Board

HUMAN SUBJECTS RESEARCH
PROTOCOL APPLICATION – Part B

1. PROTOCOL TITLE: Validation of the STarT Back Screening Tool in the Primary Care Management of Low Back Pain in the Military Health System: A Randomized Trial of Risk-stratified vs. Usual Care

2. ABSTRACT

This is a pragmatic Randomized Clinical Trial where 290 subjects will be recruited from primary care clinics within four Army Medical Centers that have a primary complaint of LBP. Subjects will complete screening questionnaires at baseline to assess all patients on key psychosocial and physical factors that have prognostic implications for predicting risk of delayed recovery. The SBST will be utilized to classify patients into one of three risk categories (low, medium or high) for targeted treatment, based on the presence of potentially modifiable physical and psychological prognostic indicators for persistent, disabling symptoms. Physical factors such as acuity and location of symptoms also have prognostic implications for predicting immediate benefit for spinal manipulation. All subjects will be assessed at baseline according to these factors, then randomized to receive risk-stratified care based on the results of the SBST and spinal manipulation screening (Risk Stratified Care) or care based on current clinical practice guidelines (Usual Care). The experimental aspect of this study is to see if the risk stratification tool will do a better job at dictating the specific type and timing of treatment provided to the patient, compared to usual care.

3. OBJECTIVES/SPECIFIC AIMS/RESEARCH QUESTIONS.

Purpose: To compare a risk-stratified approach to a usual care approach in the management of low back pain (LBP) in primary care using the recently developed STarT Back Screening Tool (SBST).

The specific aims of the study are the following:

PRIMARY AIM:
Specific Aim 1: Compare clinical outcomes of care (pain, disability, quality of life, fear-avoidance beliefs, depressive symptoms, and patient and provider satisfaction) between risk-stratified care according to the STarT Back Screening Tool and usual care approach in the management of patients with LBP in the primary care setting.

Hypothesis Specific Aim 1: Greater improvements in both short- and long-term clinical outcomes will be observed among patients treated with a risk-stratified versus a usual care approach.

SECONDARY AIMS:
Specific Aim 2: Compare direct and indirect costs associated with risk-stratified versus usual care in the management of patients with LBP in the primary care setting.

Hypothesis Aim 2: Direct and indirect healthcare costs will be lower at 12 months among patients treated with risk-stratified care versus usual care.

Specific Aim 3: Compare the cost-effectiveness of risk-stratified care versus usual care in the management of patients with acute LBP in the primary care setting.

Hypothesis Specific Aim 3: Risk-stratified care will be more cost-effective than usual care in managing patients with acute LBP in primary care.

Note: If the data analyses for the first two specific aims indicate that risk-stratified care is associated with significantly lower costs and significantly superior clinical outcomes, the treatment may be recommended and no additional cost-effectiveness analysis would be indicated. If risk-stratified care is associated with significantly greater costs and significantly inferior clinical outcomes, this treatment approach cannot be recommended and no further cost-effectiveness analysis would be indicated. However, if the costs associated with risk-stratified care are higher than usual care (or statistically not different), but the clinical outcomes are improved with risk-stratified care; a cost-effectiveness analysis to address Specific Aim 3 will be performed.

4. MILITARY RELEVANCE

Problem of LBP in the Military Health System

LBP is among the most frequent causes of medical visits and lost-duty time in the Military Health System (MHS). In 2009, LBP (intervertebral disc disorders and other disorders of the back including lumbago and unspecified backache) resulted in 606,332 outpatient medical encounters, accounting for 6.4% of all outpatient visits for any illness or injury among active component members.(3) During a 10-year period (2000-2009) and after excluding other medical conditions that may cause back pain, there were 7,008,557 ambulatory visits (among 1,020,701 individuals) and 31,675 hospitalizations (among 26,575 individuals) with mechanical LBP-related diagnoses.(4) During this same period, 7.4% of all active component members had at least one diagnosis of mechanical LBP.(4)A recent study reported that the unadjusted incidence rate of LBP was 40.5 per 1,000 person years in active duty service members with the highest incidence noted in the Army.(5) The Army, Navy, and Air Force service members had higher incidence rates of LBP than the Marines, with incidence rate ratios (using Marines as the reference 1.0) for the Army = 2.19, Navy = 1.02 and Air Force = 1.54.(5)

Musculoskeletal pain, and especially LBP, adversely affects military preparedness as common reasons for medical evacuation from ongoing conflicts (1) with return to duty being uncertain.(1)(2) Of 860,524 service members who received an initial diagnosis of LBP from 2000-2009, approximately one-fourth (23%) had at least one medical encounter for LBP within one year after the incident episode. Over 50% of all service members with at least one LBP diagnosis from 2000-2009 had at least one recurrence during the same period.(4) Moreover, from the time of their initial medical encounters for LBP, approximately one-fourth of those still in military service had at least one LBP-related encounter during each of the next nine years.(4) Only 24% of soldiers with LBP in OIF/OEF experienced at least a 50% pain reduction after treatment.(6) Back problems are leading causes of medical evacuations from Iraq and Afghanistan, with only 2% returning to combat duty.(6) LBP was the primary complaint of 53% of soldiers in OIF/OEF who presented to a military pain management center,(6) a leading cause of medical evacuation in OIF/OEF.(1)Since the beginning of military operations in those countries, more than 3,100 U.S. service members have been medically evacuated from theater due to LBP (1).

The consequences of LBP are on long term disability and costs within the MHS and VA are near catastrophic. Soldiers in the US Army have a high risk of disability 5 years after suffering low back injury.(7) In a study

among veterans treated in a VA regional healthcare network, Morasco et al(8) found that, even after controlling for numerous potentially confounding factors, LBP was significantly associated with increased risk of high-dose opioid use. Moreover, these patients frequently did not receive care consistent with current treatment guidelines. There was frequent use of short-acting opioids, and 32.0% received concurrent benzodiazepine prescriptions, which is associated with a risk for overdose and death.(8)

5. BACKGROUND AND SIGNIFICANCE.

Background: Low back pain (LBP) is among the most frequent causes of medical visits and lost-duty time in the Military Health System (MHS). In 2009, LBP (intervertebral disc disorders and other disorders of the back including lumbago and unspecified backache) resulted in 606,332 outpatient medical encounters, accounting for 6.4% of all outpatient visits for any illness or injury among active component members. Musculoskeletal pain, and especially LBP, adversely affects military preparedness as common reasons for medical evacuation from ongoing conflicts(1) with return to duty being uncertain.(2)The consequences of LBP on long term disability and costs within the MHS and VA are near catastrophic.

Key Points:

- A relatively small percentage of patients with LBP will develop chronic disability, however these individuals account for a disproportionate share of healthcare expenditures.
- Most clinical practice guidelines recommend only advice and education for all patients with non-specific LBP during the initial weeks of management, with consideration of psychosocial factors and referral to physical therapy recommended only when recovery is delayed.
- Psychosocial factors have been identified as risk factors that act as “obstacles to recovery” and increase the risk of developing chronic disability. Evidence also suggests that patients with symptoms for less than 2 weeks and who do not have symptoms distal to the knee preferentially benefit from early spinal manipulation. Therefore targeted management strategies (risk-stratified care) initiated immediately upon initial consultation in primary care may be more cost-effective than delaying treatment (usual care) for some patients.
- Recent research has demonstrated that the STarT Back Screening Tool is useful for classifying patients as being at low, medium, or high risk for experiencing chronic, disabling LBP. Targeted interventions for patients in each risk subgroup have also been developed to address the specific modifiable prognostic indicators identified by the tool. Patients who received stratified care utilizing the STarT Back Screening Tool demonstrated greater changes in disability, increased quality of life, and lower healthcare costs compared to patients in the control group at 12 months.
- One of the limitations of the STarT Back trial’s study design was that there was no standardization of the physical therapy interventions that were delivered. Therefore, it’s difficult to ascertain whether the favorable outcomes in the risk stratified group are attributable to superior physical therapy intervention or the overall effectiveness of the stratification process in directing the right patients to physical therapy with or without psychological augmentation.
- It is also unknown whether a similar stratified care approach will achieve similar results in the primary care management of patients with LBP in the MHS.
- If these results could be validated, this simple-to-use screening strategy could be implemented practically and efficiently across the MHS, with the expectation that the MHS would realize substantial cost savings and lower disability among MHS beneficiaries with LBP. Therefore, the purpose of this study is to validate the clinical and cost effectiveness of the STarT Back Screening Tool in the primary care management of patients with LBP in the MHS.

Management of Low Back Pain in the Military Health System

The majority of patients with LBP initially access healthcare through a primary care manager (9),(10). In fact, next to the common cold, LBP is the most common symptomatic reason for a primary care visit in the United States (11). Given the volume of patients with LBP managed in primary care, decisions in this setting have substantial implications on the subsequent course of a patient's symptoms and implications for overall healthcare cost (12). The joint Department of Defense (DoD) and Veteran's Health Administration (VHA) Clinical Practice Guideline for the Management of Low Back Pain or Sciatica in the Primary Care Setting was published in 1999 to optimize the management of LBP within the MHS.(13) More recently in 2008, given the similarities in evidence-based recommendations for the primary care management of LBP in both the civilian and military practice settings, the DoD-VHA LBP Working Group recently adopted the LBP practice guideline published by the American Pain Society/American College of Physicians clinical practice guideline in 2007(14) as its own. Results of LBP studies in primary care(15)-(16) have demonstrated that treatments such as manual therapy, exercise, and cognitive behavioral approaches are more effective than usual care for LBP. However, because of insufficient evidence, clinical practice guideline recommendations(17) are inadequate to inform the clinical selection of patients who are likely to benefit from additional interventions that might require referral.

Defining optimal primary care management of patients with LBP has proven elusive, and wide variations in primary care practice have been observed for decisions such as prescribing medication, ordering imaging, and referral to specialists (18),(19). Initial referral decisions for the majority of patients with non-specific LBP are based on clinical intuition, despite evidence to suggest that such a strategy provides inefficient and inconsistent access to treatment (20). Recent studies examining health care utilization trends in the United States demonstrate increasing rates of use for epidural injection procedures,(21),(22) surgeries, particularly with fusion,(23),(24) and opioid pain medications among individuals with LBP (19). In a recent survey of primary care managers, Williams et al(25) found that although guidelines discourage the use of imaging, over 25% of patients were referred for imaging. Patients were also frequently prescribed nonsteroidal anti-inflammatory drugs and opioid medications, despite recommendations that initial care be focused on giving advice and simple analgesics. Despite increasingly aggressive treatments, there is no evidence that clinical outcomes are improving; in fact, rates of chronicity related to an episode of LBP are increasing (26),(27).

Alternatively, a one-size-fits-all primary care strategy(28) that refers all patients with LBP for treatment is also generally thought to be suboptimal because it ignores the heterogeneity in patients,(29) resulting in an impractical and inefficient system because of high numbers and costs (17),(30),(31). For example, clinical practice guidelines mostly recommend delaying referral to physical therapists for at least 4 weeks following initial primary care consultation (32),(33). This approach is based on the belief that most patients with LBP will recover rapidly, and intervening quickly would not be cost-effective (34). It is further believed by some that early intervention may impede recovery for some patients by excessively “medicalizing” the condition (35-36). However, this is contrasted by evidence suggesting that the implementation of evidence-based interventions by a physical therapist, for example, earlier in the course of care may prove more cost-effective by promoting recovery and reducing the need for more invasive and costly interventions. Research examining the outcomes of primary care management for patients with LBP supports this concern, with studies indicating a majority of patients go on to experience persistent and/or recurrent symptoms, and up to one-third report moderate to severe pain one year following the initial primary care encounter (36-37).

A novel approach gaining interest in other medical specialties is to determine whether stratified care according to the estimated risk of poor prognosis improves clinical outcomes. Researchers at Keele University in the United Kingdom recently developed a risk stratified model for back pain, which consists of two complementary components. First, they utilized a simple screening method referred to as the STarT Back Screening Tool (SBST), which classifies patients into one of three risk categories (low, medium, and high) for targeted treatment based on the presence of modifiable physical and psychological indicators of persistent, disabling

symptoms. Patients are classified as "low risk" of developing future disabling back pain if they score positively on fewer than 4 questions. The remainder are then subdivided into "medium risk" (physical and psychosocial indicators for poor outcome, but without high levels of psychological indicators) and "high risk" (high levels of psychological prognostic indicators with or without physical indicators). Targeted interventions for patients in each risk subgroup have also been developed. In a recent trial to test its effectiveness, patients who received stratified care experienced significantly greater changes in disability compared to patients who received usual care at 4 months. Moreover, at 12 months, stratified care was associated with a mean increase in quality-adjusted life years (QALYs) and cost savings.

Chronic disability related to LBP within the MHS imposes a tremendous economic burden to the health care system and a burdensome amount of pain and suffering to afflicted individuals. This study has high impact potential because it proposes to tests the validity of a previously developed triage model for back pain that works in the civilian population in the MHS beneficiary population. This study would be the first clinical trial implementing a risk-stratification decision point from which to triage patients with back pain in the MHS. Essentially, a very important study that will let a clinician know which patients will be ok with only minimal care, and which patients need additional early care to help prevent their condition from turning chronic. The results could help standardize care across the MHS, and help direct patients down an individualized management pathway that is most likely to benefit them. The results of this study will improve the quality of care within the MHS by establishing standardized clinical practice guidelines for the most common musculoskeletal injury seen in the MHS. Health policy will be informed regarding the optimal care delivery systems for these patients. Improving referral patterns for specialty care related to back pain, and adherence to practice guidelines presents an important opportunity to improve the cost-effectiveness and quality of care related to managing back pain within the MHS.

6. RESEARCH DESIGN

The proposed study will be a pragmatic randomized clinical trial with a 1-year follow-up period. The trial will compare two approaches to the management of LBP in primary care. The Usual Care group will receive treatment based on current evidence-based practice guidelines advocating initial management of education and advice to remain active for all patients with non-specific LBP, with more intensive interventions reserved for patients who fail to recover. Patients in the Risk Stratified group will be further sub-grouped based on their risk of developing chronic symptoms, and need for physical therapy with or without cognitive behavioral principles. Patients will be stratified into low, medium or high risk level according to the STarT Back Screening Tool, and treatment will be tailored based on those risk levels.(38)

7. RESEARCH PLAN

7.1 Selection of Subjects Patients who are consulting primary care for an initial episode of low back pain

7.1.1. Subject Population. We will enroll 290 consecutive patients seen in primary care with a primary complaint of LBP who consent to participation. Patients will be enrolled in the primary care clinics at four Army medical centers. We will plan to enroll between 90 and 140 subjects at the SAMMC site, as this will be the primary site. We will start off with a lower target, but if the other sites under-enroll, then we will enroll up to 140, but will ensure that total enrollment does not go over 290 between all 4 sites. The goal will be to enroll from 60 to 80 subjects at each of the 3 other sites, but again ensuring that enrollment across all sites does not go over 290. If one site under-enrolls, the other sites will enroll up to 80 subjects (except BAMC, will enroll up to 140).

7.1.2. Source of Research Material.

Source of Research Material	Clinical Purposes(Y/N)	Research Purposes (Y/N)
Self-report function, disability, risk screening, and quality of life questionnaires<br>1. Demographics<br>2. Pain Body Diagram<br>3. Quality of Life - EuroQoL (EQ-5D)<br>4. Promis-29<br>5. Credibility Expectancy Questionnaire (CEQ)<br>6. STarT Back Screening Tool (SBST)	Y	Y
Biopsychosocial Measures<br>1. OSPRO-YF	Y	Y
Healthcare Utilization (MDR Healthcare Database)<br>1. Imaging<br>2. Medication prescription<br>3. Specialty referrals & visits<br>4. Medical procedures	N	Y

7.1.3. Inclusion and Exclusion Criteria.

<u>Inclusion criteria:</u>

The following inclusion criteria will be used to determine eligibility for the study. Potential candidates must satisfy all criteria and consent to participation to be eligible.

1) Males and females who are between the ages of 18-50 years old
2) Primary complaint of LBP for any duration, with or without associated radiculopathy
3) Can speak and understand English
4) Be willing and able to give full, informed written consent

<u>Exclusion criteria:</u>

1) Any potentially serious disorders (e.g., cauda equina compression, inflammatory arthritis, compression fracture, malignancy, infection), serious illness, or comorbidity
2) Spinal surgery in the past 24 months
3) Current pregnancy (or within the last 6 months)
4) Already receiving treatment (other than primary care) for this episode of LBP
5) Inability to attend regular treatment sessions
6) Pending litigation or a medical evaluation board

7.1.4. Description of the Recruitment and Prescreening Process. Providers from the identified primary care clinics (i. e., Fort Sam Houston Primary Care Clinic and Schertz Medical Home) will alert research staff when they have patients on their roster with a primary diagnosis of low back pain. We have discussed this approach with the Chief of Primary Care. The majority of patients book an appointment within 72 hours of the appointment time, taking use of online booking features. Therefore clinicians and research staff will be able to see a roster of patients with a reason of “back pain” listed. A HIPAA waiver has been requested in order that approved research staff can review the list of patients coming into the clinic that day with a diagnosis of low back pain. No additional laboratory or examination findings will be necessary. Because of the nature of the intervention, which will vary based on the treatment group the patient is randomized to, the decision to enroll in the study needs to be made early in the medical appointment process. Therefore, the reason for the appointment (back pain) will be used as the pre-screening criteria.

7.1.5. Subject Screening Procedures.
Patients that are presenting with LBP, as identified in the prescreening process, will be approached by a member of the research staff regarding their interest in participating in this study. This will occur while they are waiting to see their PCM, as there is often 10-15 minutes of waiting time after they have checked in before the patient is called back. The Primary Care clinics will contact the research team regarding upcoming appointments that are booked for back pain. At that point, members of the research team (all who are credentialed doctors of physical therapy), will ensure they are there to approach the patient when they arrive. There is often 15-20 minutes of time from when the patient checks-in to when they are seen by the PCM, so the Primary Care leadership has agreed that this is an opportune time. We are currently enrolling subjects from the Primary Care clinic for a different study and this method has proved very efficient and highly supported by the Primary Care Staff. The PCM can also put potential participants in touch with the Study Coordinator, to explain the study in more detail and provide further information about it. Patients will be assured that participation in the study is completely voluntary and that declining to participate will in no way impact the patient’s medical care. All patients will be referred for an assessment by an experienced physical therapist who will work at the clinic, regardless of whether they choose to take part in the trial or not. Patients coming with LBP will be identified by the front desk clerks, who will in turn notify the research team.

The member of the research staff will approach the patient with the following script:

“Hello Mr/Mrs [Name}, my name is {their name} and I’m helping conduct a research study that is assessing how we can best initially manage patients with low back pain. Would you be interested in hearing more information about our study related to early management of back pain? It will not affect your ability to receive care, should you choose not to participate.”

If they agree, they will be consented by an approved member of the research study in a private setting, in order to determine if they meet the inclusion/exclusion criteria.

7.1.6. Consent Process.
Patients identified through the prescreening process will be approached during check-in regarding the option of participating in a study related to how patients are managed for low back pain. If they are interested, they will be taken into a separate room and an approved member of the research team will explain the nature and purpose of the study to the patient, to include: follow-up time points, requirements, nature and type of data collected (future healthcare utilization to include additional referrals, medicine, imaging, etc related to their back pain), etc. If the patient is interested in participating, the research team member will review the informed consent document with the patient and then obtain their consent. They will then screen the patient to make sure they meet the inclusion/exclusion criteria for involvement in the study.

7.1.7. Compensation for participation. None

7.2 Drugs, Dietary Supplements, Biologics, or Devices.

7.2.1 N/A

7.2.2 N/A

7.3. Study Procedures/Research Interventions.

Randomization:
After completion of baseline assessments, subjects will be randomized into one of two arms (Group I = Usual Care, Group II= Risk Stratified Care based on the SBST). The method of group assignment will be sequentially numbered opaque sealed envelopes (SNOSE). To minimize the risk of predicting the treatment assignment of the next eligible patient, randomization will be performed in permuted blocks of two or four with random variation of the blocking number. Of the 290 subjects expected for enrollment, ~140 (48%) are expected to be enrolled at BAMC, and ~50 (17%) to be recruited at the other 3 sites. Randomization envelopes for subjects 141 to 290 will be sent to other sites, but will also be coded locally for tracking purposes. For example, subject 141-190 at Madigan will be M1-M50, 191-240 at Womack will be W1-W50, and 241 to 290 at WBAMC will be FB1-FB50 (Fort Bliss). Because we are using permuted block randomization, there will be a balanced number of each test group for each site.

Management of Patients in the Usual Care Group: Clinical practice guidelines recommend an evidence-based physical examination that limits the use of diagnostic imaging except when serious pathology (ie, "red flags") is suspected.(39) Current clinical practice guidelines also recommend an active management approach that emphasizes the importance of remaining active, and education on the favorable prognosis of LBP.(14) Therefore, patients in both groups will receive a structured, 30-minute history and physical examination consistent with practice guidelines. The examination will include a screen for potential serious pathology ("red flags") and neurological examination (lower extremity reflexes, sensation and muscle strength). Patients will be asked about their symptom history, concerns, and treatment expectations. A brief physical examination will be performed that assesses back movements, including testing for a directional preference among patients with potential radiculopathy, which will be defined as having symptoms distal to the buttocks. A screening examination for relevant hip involvement will also be performed. Diagnostic imaging will be at the discretion of the PCM for patients in the Usual Care group but limited to those patients for whom serious pathology is suspected in the Risk Stratified group.(39)

Patients in both groups without "red flags" will also receive an active management approach as currently recommended in clinical practice guidelines.(14) This approach emphasizes positive information and advice and focuses on encouraging and supporting patients in early activity, minimizing bed rest, emphasizing a favorable prognosis, promoting appropriate levels of activity, encouraging return to work, and engendering positive attitudes to pain.(14) These principles will be reinforced by the patient's PCM and associated practice staff. PCMs will limit medication prescriptions for both groups to a small range of drugs (analgesics, non-steroidal anti-inflammatories, etc.) according to clinical practice guidelines.(40) Regardless of treatment group, participants will not be restricted from using health care elsewhere or seeing their PCM during the follow-up.

Upon completion of the structured examination and active management intervention, patients in the Usual Care group will be scheduled for a follow-up appointment with their PCM four weeks after the baseline examination. Patients will be instructed that they can call to schedule an earlier follow-up if they feel that it is necessary. The results of the baseline STarT Back Screening Tool score will not be revealed to the PCM at

any point during the patient's participation in the study. Therefore, decisions about ongoing referral to further physical therapy or other specialty care will be based on clinical need according to the PCM's clinical judgment, without knowledge of a participant's STarT Back Screening Tool classification score. In the event that a referral to physical therapy is made, no specific guidance will be provided on the number of sessions or length of treatment. It is highly unlikely that the PCM for patients in the Usual Care group will object to being blinded to the STarT Back Screening Tool score because this tool is not currently the standard of care and is not mentioned in clinical practice guidelines.

Ongoing Physical Therapy According to Treatment-Based Classification Principles

For patients in the Usual Care group who are referred to physical therapy at the discretion of the PCM, and for patients in the Risk Stratified group who are referred to physical therapy based on the STarT Back Screening Tool score, all physical therapy will be delivered according to treatment-based classification (TBC) principles.(41) Consistent with TBC principles, a tailored management plan will be devised based on the patient's history and physical examination that includes using evidence-based treatments such as advice and explanation, reassurance, education, manual therapy, strengthening exercise, directional preference exercise, and traction. These sessions will be individualized treatments lasting 30-60 minutes and will be held in the local outpatient physical therapy clinic located in close proximity to the primary care clinic. The sessions will be focused on reducing back-related disability, restoring function, and targeting physical characteristics (disabling back pain, referred leg pain, and co-morbid pain). During the first session, patients will be re-assessed, including a detailed differential diagnosis, particularly for patients with referred leg pain (ie, potential radiculopathy). Consistent with evidence-based guidelines,(14) treatments such as bed rest, massage, and electrotherapy will not be included in the treatment protocol. Treatments will be provided by physical therapists who already routinely provide care to patients with LBP and who have been specifically trained in TBC principles. This is standard of practice for many physical therapists, but the focus will be reinforced to all. We will use a management protocol that we have used in prior LBP research.(42)

Management of Patients in the Risk Stratified Group
Similar to the Usual Care group, all patients in the Risk Stratified group will receive a 30-minute structured history and physical examination and initial treatment according to active management principles as previously described. The primary difference is that the active management strategy will be enhanced with a more standardized delivery (Table 1). For example, patients will receive reassurance to address specific concerns related to their LBP and any resulting loss of function or implications on their work. Reassurance topics will be guided by the results of the patient's STarT Back Screening Tool score so that specific concerns can be identified and addressed on an individual basis. Messages of advice will focus on:

- Appropriate levels of activity including return to work (if appropriate) and avoiding bed rest
- Patients will be given a pamphlet about local exercise venues and self-help groups and view a 15-minute educational video entitled "Get Back Active"
- Patients will be given the "*Back Book*",(43) a patient information booklet shown to positively affect patients' understanding of active management principles.(44)(45) Its development incorporated content from two previous booklets shown to alter beliefs and outcomes in patients with LBP.(46)(47) The primary purpose of the *Back Book* is to improve fear-avoidance beliefs about back pain rather than imparting factual information regarding appropriate posture, lifting strategies, etc.(44) The *Back Book* is well accepted by patients and has been shown to improve clinical outcomes and shift beliefs in a positive direction compared to traditional education based on the biomedical model.(44) Importantly, these improvements were observed despite primary care staff's minimal investment of time reinforcing these principles and monitoring adherence, suggesting the *Back Book* works well in the context of a busy primary care setting.

A copy of the *Back Book* will be provided to a patient immediately after being randomized to the Risk Stratified group. The contents and major messages of the pamphlet will be reinforced verbally by the PCM or Research Physical Therapist. Members of the primary care team and treating physical therapists will be trained in these principles and will be responsible for reinforcing these messages throughout the patient's course of care. Patients will be asked to read the booklet upon returning home from their primary care office visit, and the principles will be reinforced during the initial physical therapy session for patients in the medium and high risk groups.

- Addressing patient fears supported by the *Back Book (http://www.nrmc.co.uk/wp-content/uploads/The-Back-Book.pdf)*
- Addressing an individual's uncertainty about issues such as use of pain relief (medication), the role of further investigations, work issues, and the patient's likely future prognosis including methods to deal with future episodes of LBP.

**Risk Stratified Care**	**Usual Care**
**Low, medium and high risk groups**<br>-Evidence-based assessment of LBP according to current clinical practice guidelines, to include limited use of imaging except for patients with "red flags"<br>-Enhanced active management advice emphasizing positive messages about activity, pain relief and work for LBP<br>- Reassurance to address specific concerns related to their LBP and implications on work<br>-Patients given a copy of the *Back Book* and see a 15-minute video based on the *Back Book* entitled "Get Back Active"	**All patients**<br>-Evidence-based assessment of LBP according to current clinical practice guidelines, to include limited use of imaging except for patients with "red flags"<br>-Active management advice emphasizing positive messages about activity, pain relief and work for LBP<br>-No specific guidance regarding physical therapy referral, thus decision to refer or not will be made by the PCM according to usual practice<br>-Physical therapy according to TBC principles if a referral to physical therapy is made
**Low risk group**<br>-The above PLUS two-item spinal manipulation screening, with spinal manipulation delivered in primary care if indicated<br>-No referral for ongoing physical therapy	
**Medium risk group**<br>-Same as low risk group PLUS<br>-Patient is referred for ongoing physical therapy based on the Treatment Based Classification principles for up to 8 visits, 30-60 minute sessions (twice weekly)	
**High risk group**<br>-Same as low and medium risk group PLUS<br>-Patient is referred for ongoing physical therapy using TBC principles for up to 12 visits, 45-60 minute sessions (twice weekly)<br>-Physical therapy is psychologically augmented with the assessment of biopsychosocial risk	

factors and the adoption of cognitive behavioral principles that explore patient concerns and address unhelpful beliefs and behaviors. These strategies will include tailored education, graded exercise, graded exposure, etc.	
**Table 1. Components of risk stratified care vs. usual care**	

Based on the results of the STarT Back Screening Tool score, patients in the Risk Stratified group may receive no additional intervention beyond usual care, or may receive additional physical therapy intervention, with or without cognitive behavioral principles. Therefore, unlike patients in the Usual Care, group, the STarT Back Screening Tool scores in the Risk Stratified group will be revealed to the PCM immediately after randomization, and any additional care that is indicated will begin shortly thereafter. One of the other primary differences is that for patients in the Risk Stratified group, PCMs will be encouraged to adhere to recent clinical practice guidelines that recommend limiting referrals to specialists other than the physical therapist (ie, orthopaedist, neurologist, physiatrist, psychiatrist etc.), except in cases where the PCM suspects an emergent condition that requires immediate intervention. Referrals to specialty care in the Usual Care group will not be limited in any way. In the end it will be up to the individual preference of the PCM what management decisions are made, but they will have this additional information to help better inform their decision.

Risk Stratification Treatment Algorithm

Low risk-group

Patients allocated to the “low risk-group” according to the STarT Back Screening Tool will receive care limited to the initial clinic session as previously described and reassured that further treatment is unlikely to be beneficial or necessary. These patients will not be encouraged to seek further treatment. They will be advised, however, that if their symptoms worsen, they should re-visit their PCM. In addition to the initial structured examination and enhanced active management intervention, patients in the low risk group will also be examined according to a two-item spinal manipulation screening (48). Previous research has demonstrated that a subgroup of patients with acute LBP are likely to experience more rapid, pronounced and lasting reductions in pain and disability with an early intervention of spinal manipulation and exercise. The two items in the screening are the duration (in days) of the patient’s current episode of LBP, and whether or not the patient has experienced symptoms extending below the knee during the current episode. When symptom duration is fewer than 16 days and no symptoms extend distal to the knee, the patient is considered highly likely to benefit from an early manipulation intervention. Patients who are categorized as good manipulation candidates will receive a single session of spinal manipulation using a standardized technique, if they consent to the treatment. To perform this technique, the patient will be supine. The therapist stands opposite the side to be manipulated and side-bends the patient away from the therapist. The patient interlocks the fingers behind the head. The therapist rotates the patient, and delivers a quick thrust to the anterior superior iliac spine in a posterior/inferior direction (Figure 1). After the manipulation, the therapist notes whether a cavitation (a “pop”) was heard or felt by the therapist or patient. If a cavitation is noted, the therapist will proceed to instruct the patient in a basic range of motion exercise to be performed at home. If no cavitation is noted, the patient will be repositioned, and the manipulation will be attempted again. If no cavitation occurs on the second attempt, the therapist will manipulate the opposite side. A maximum of 2 attempts per side is permitted. If no cavitation is produced after the fourth attempt, the therapist will proceed to instruction in the range of motion exercise. The range of motion exercise to be performed will be a supine pelvic tilt exercise. Patients will be instructed to perform 10 repetitions in the clinic and 10 repetitions of the exercise 3-4 times daily at other times throughout the day. If the patient prefers not have a treatment of spinal manipulation, this will be noted, and the patients will do the exercises only. These patients will be discharged from further physical therapy care at the end of the initial clinic consultation. They can follow-up with their primary care provider at any time for further care if they feel the need.

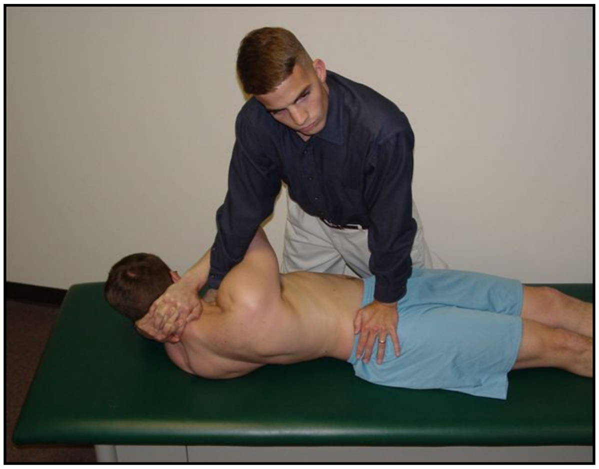
Figure 1: Spinal manipulation technique

Medium Risk Group
Patients allocated to the “Medium risk-group” according to the STarT Back Screening Tool will receive the same care as those patients in the low risk group, plus all patients in this category will be referred for ongoing physical therapy using TBC principles as previously described for up to 8 visits (twice weekly), with each session lasting 30-60 minutes. Moderate levels of psychological prognostic indicators will be addressed, but specific training on techniques to target psychological factors will not be provided for physical therapists treating the medium risk-group of patients. Therapists will be advised to refer non-responders on for further investigations or secondary care interventions, with supervision provided if required from a physical therapist who specializes in the management of LBP.

High Risk Group

In addition to the first clinic session described above, patients allocated to the "high risk group" according to the STarT Back Screening Tool will receive the same care as those patients in the low and medium risk groups, plus all patients in this category will be referred for ongoing physical therapy using TBC principles as previously described for up to 12 visits (twice weekly), with each session lasting 30-60 minutes.

The focus of these sessions will be on restoring function using combined physical and psychological approaches and targeting physical and psychological obstacles to recovery. Physical therapy will be psychologically augmented with the assessment of biopsychosocial risk factors and the adoption of cognitive behavioral principles that explore patient concerns and address unhelpful beliefs and behaviors. These strategies will include tailored education, graded exercise, graded exposure, etc. Therapists will use "stem & leaf" questions to identify unhelpful beliefs and behaviors. Physical treatment modalities (exercise and manual therapy) will be integrated with psychologically informed techniques to provide a credible explanation for symptoms, reassurance, education, collaborative goal setting, problem solving, pacing, graded activity, and relaxation. There will be a specific focus on the prognostic psychological indicators identified by the STarT Back Screening Tool such as low mood, anxiety, pain-related fear and catastrophizing. Reasons for psychological distress will be addressed using enhanced communication skills with a focus on promoting appropriate levels of activity, return to normal activities, and the management of future back pain recurrences. Patient expectations about prognosis and implications for function will be addressed and the role of active self-management emphasized. Advice about sleep and work will be provided and if necessary a return to work plan implemented. Patients will be encouraged to put management plans into practice between treatment sessions, and help will be given to problem solve any difficulties that arise. Monthly group mentoring sessions will be held for physical therapists to discuss individual cases and consolidate the training throughout the trial, with supervision provided from a Consultant Physical Therapist (pain management expertise). Therapists will be advised to refer non-responders on for further investigations or secondary care interventions.

Patients in the Risk Stratified group who are categorized in the high risk group will receive care based on TBC principles but augmented with cognitive-behavioral principles. Exercises for patients receiving the cognitive-behavioral intervention will be similar to those used in previous studies showing the benefits of physical therapy using cognitive-behavioral principles.(49)1, (50) Two types of exercise will be used: general and patient-specific. General exercise will consist of low stress aerobic activity (e.g., treadmill, stationary cycle, etc.) and strengthening of large muscle groups. Patient-specific exercises will be determined by the physical therapist based on the TBC principles,(41) and using previously published exercise protocols (42). The physical therapist will examine each patient for the presence of a directional preference that would indicate the need to perform specific exercises in a certain direction. A directional preference occurs when either a posture or repeated movement in one direction (e.g., flexion, extension, etc.) decreases or abolishes low back pain, or cause referred pain from the spine to appear to progressively retreat in a proximal direction back toward the lumbar midline ("centralization").(51) When a directional preference is present, prescribing repeated, end-range exercise in the direction of directional preference has been shown to result in superior short-term clinical outcomes (51). Patients will also receive trunk strengthening and stabilization exercises, which may reduce the risk of recurrence (52). Exercises will be used targeting the primary stabilizers of the lumbar spine including the oblique and transverse abdominals, erector spinae and multifidus muscles(53). Finally, the patient will be asked to identify tasks or activities that they perceive to be difficult or are fearful of performing due to their back pain. The therapist will design exercises to imitate the task. For example, a patient who reports difficulty, or is fearful of lifting groceries from the trunk of a car might be given an exercise to practice this action using sound body mechanics with a certain number of repetitions.

Each physical therapy session will last about 30-60 minutes. The patient will perform their exercises during each session. Consistent with a cognitive-behavioral approach, the emphasis during physical therapy sessions

will be on attainment of exercise goals instead of pain. Patients will be reassured that discomfort during usual physical activity and exercise does not indicate structural damage to the spine. Physical therapists will be trained to understand the fear-avoidance model, reminding patients at each treatment session that LBP is a common condition, not a serious disease. Patients will also be frequently reminded that their prognosis is favorable, reinforcing the positive information and advice they received in primary care. The relationship between pathoanatomy and LBP will be de-emphasized. Patients will be encouraged to take an active role in their recovery, thus the use of passive treatment modalities such as superficial heat, therapeutic ultrasound, etc. will be avoided.

During the first physical therapy session, the treating physical therapist will determine whether the patient has reviewed the *Back Book* (44-45) provided to them in primary care. If the patient indicates the educational pamphlet was read, the treating physical therapist will briefly review the pamphlet's contents with the patient. If the pamphlet was not read, the therapist will ask the patient to read the pamphlet during the first treatment session, after which the therapist will review the contents with the patient to determine if the patient understands the material or has any questions.

The progression of exercises in the cognitive-behavioral intervention will not be based on the patient's symptomatic response, but instead graded exercise principles will be used in an attempt to direct attention towards attaining certain functional goals and away from the symptom of pain (49). Graded exercise prescription has been recommended for patients with elevated fear-avoidance beliefs and has been shown to have favorable effects on return to work rates compared to usual care (54-55). The hallmark of graded exercise is that exercise prescription is based on achieving a predetermined quota of a specific exercise intensity, duration of exercise, or number of repetitions,(56) as opposed to cautioning patients only to exercise within a pain-free range of movement.

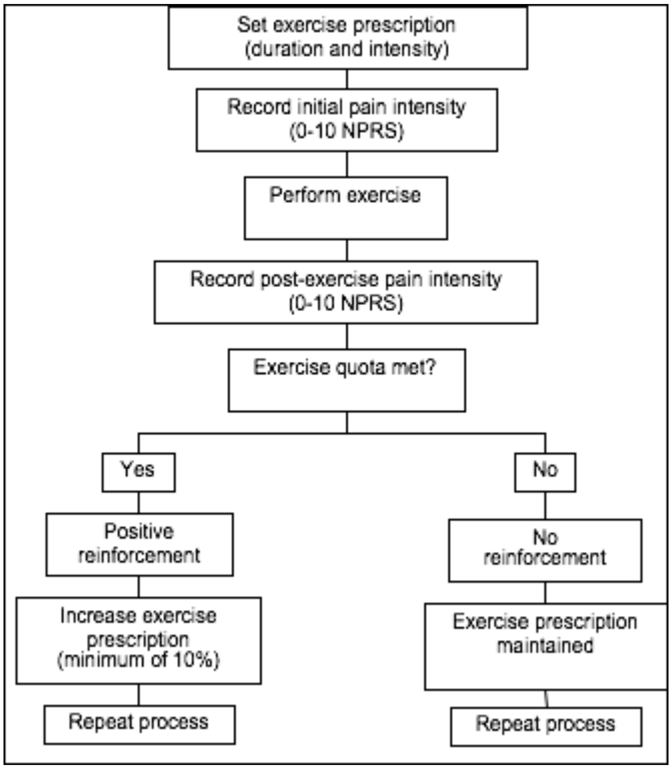

Figure 2: Exercise Prescription Process

The initial number of repetitions or duration of exercise will be determined by the treating physical therapist, representing the baseline exercise quota. Attempts will be made to set the initial exercise quota at a level that does not increase the patient's pain intensity.(57) When the quota is reached, the patient will receive positive reinforcement, and the physical therapist will establish a new exercise quota that is a minimum 10% increase compared to the previous quota. Examples of positive reinforcement that will be used include verbal encouragement, a brief rest period, etc. Patients not achieving the established exercise quota will be given another opportunity to reach their exercise quota at the next treatment session (Figure 2). Pain intensity will be measured before and after exercise but will not be used to make decisions about exercise progression. An exception to the quota-based system is if a patient experiences a worsening of their baseline symptoms (i.e. peripheralization of symptoms) during the prescribed exercise. In this case, the patient's exercise will be discontinued, and the physical therapist will re-examine the patient to determine the most prudent future course of care according to TBC principles (41). Patients will also be gradually exposed to specific activities they consider potentially painful or difficult to perform (46). Compliance with the home exercise program and specific exercise progression sequences will be recorded on the exercise log, as per standard of care.

**Assessment**	**Visit / Follow Up (F/U) Interval**			
Study Day / period	Same Day	6 weeks	6 months	1 Year
Screening	X			
Informed Consent, discuss Plan, etc.	X			
Randomization	X			
Demographics, History & Physical	X			
Treatment	X	X		
Promis-29	X	X	X	X
OSPRO-YF	X	X	X	X
Credibility Expectancy Questionnaire	X	X	X	X
Medication use & healthcare resource utilization (part of the Data Collection F-U Form)	X	X	X	X
STarTBack Tool	X	X	X	X
Healthcare Utilization (MDR database) for imaging, medication prescription, and specialty referral				X
Quality of Life: *EuroQoL* (EQ-5D)	X	X	X	X

7.3.1 Collection of Human Biological Specimens. N/A

7.3.1.1 Laboratory evaluations and special precautions. N/A

7.3.1.2 Specimen storage. N/A

7.3.2 Data Collection.

<u>Baseline Examination Procedures</u>

All patients who meet the inclusion/exclusion criteria and provide informed consent will receive a standardized baseline examination to be performed by the Study Coordinator who will remain blind to the patient's treatment group assignment throughout the study. The baseline examination will consist of patient-completed questionnaires. Demographic information, measures of general health, pain, disability, quality of life measures, and treatment preferences will be collected at baseline, 6 weeks, 6 months, and 12 months. Cost data will be collected at 12 months. The 6 week follow-up will be collected in person. The 6- and 12-month follow-up will be collected by regular postal mail or email with ability to submit de-identified responses electronically. For those individuals who do not return the surveys, a phone call will be made as a reminder with a voicemail if necessary.

Telephone Script:
"Good morning/afternoon Mr./Mrs. [Name], my name is [Name] and I am involved in the research study that you joined related to the care you are receiving for your low back pain. As we discussed in our original consent, we would be contacting you 6- months and 12-months after your initial participation, to see how you are feeling, by answering a few surveys. The surveys have been sent to your email address that you provided and should only take a few minutes to complete. Is it still ok that we contact you about this now? Is the email you provided still a good way to reach you? "

Voicemail Script:
"Good morning/afternoon Mr./Mrs. [Name], my name is [Name] and I am calling in regards to the back pain research study. Please give us a call back at your earliest convenience"

Self-Report Measures

STarT Back Screening Tool: The STarT Back Screening tool (58-60) is a simple-to-use prognostic screening method designed to triage the primary care management of LBP.(61), (58) The instrument classifies patients into one of three risk categories (low, medium, and high) for targeted treatment, based on the presence of potentially modifiable physical and psychological prognostic indicators for persistent, disabling symptoms, identified through 9 questions. Patients are classified as "low risk" of future disabling LBP if they score positively on fewer than 4 questions. The remainder are then subdivided into "medium risk" (physical and psychosocial indicators for poor outcome, but without high levels of psychological indicators) and "high risk" (high levels of psychological prognostic indicators with or without physical indicators). Targeted interventions have been developed for patients in each risk subgroup to address the specific modifiable prognostic indicators identified by the tool. In collaboration with clinical experts, a consensus-based method was used to develop evidence-based assessment and treatment approaches for patients with LBP according to a "stepped-care" format in which the intensity and focus of the treatment approach is "stepped up" based on risk of chronicity according to the STarT Back Screening Tool.(61) The focus of the interventions is directed towards the secondary prevention of disabling back pain.

Demographic Information: Patients will self-report a variety of demographic and descriptive information including age, gender, ethnicity, job title, employment history, and current employment status.

Credibility Expectancy Questionnaire – the CEQ, a 6-item self-report evaluating treatment credibility and expectations for improvement,(62) will be assessed at baseline after treatment group assignment is revealed to provide descriptive information about participants' perceptions of their treatment assignment and optimism for

improvement. The CEQ may be used as a covariate if perceptions differ meaningfully between groups as participants' initial perceptions of treatment credibility can impact outcomes.(63)

Disability Related to LBP

PROMIS-29 - We will use the Patient Reported Outcomes Measurement Information Systems (PROMIS) 29-item short form (version 2) (69). The PROMIS 29-item short form efficiently assesses several outcomes important to patients with low back pain; including, pain intensity and interference, sleep disturbance, anxiety, depression, fatigue, and social role participation using items developed with rigorous methodology and patient input.(70) The PROMIS-29 has previously been used as an outcome measure assessing the above domains for those with low back pain receiving physical therapy (71). The MCID is still being investigated, but found to be around 6 points in other musculoskeletal pain populations. Other studies have used the Oswestry Disability Index and the Roland Morris Disability Questionnaire, however utilization of both of these measures, in addition to other measures of sleep (Pittsburg Sleep Quality Index), and psychosocial issues creates additional burden for subjects filling out a lot of questionnaires. The PROMIS-29 is a new tool endorsed by the National Institutes of Health that encompasses all of these constructs within a shorter document for each subject.

Location and Intensity of LBP:
Patients will also complete a pain body diagram to identify the location and nature of symptoms.(72)

Quality of Life
EuroQoL (EQ-5D) – the EQ-5D is a generic quality of life questionnaire(73) that will assess quality of life on a scale that can be referenced to other disease conditions.(74) The EQ-5D covers 5 domains: mobility, self-care, usual activities, pain/discomfort, and anxiety/depression. Each domain has 3 response categories: level 1, "no problems"; level 2, "some problems"; and level 3, "inability or extreme problems." Responses are combined to give a 5-digit descriptive health state classification (e.g., 11222). The EQ-5D yields a total of 243 possible health states. Valuations for each health state are available.(75) The EQ-5D is commonly used in economic evaluation of interventions and cost-effectiveness analysis.

Biopsychosocial Measures
Biopsychosocial factors such as depression, fear-avoidance beliefs, fear of pain, and anxiety have been identified as risk factors that act as "obstacles to recovery", playing a significant role in determining those who will develop chronic pain and disability.(76) In particular, depressive symptoms have been identified as an important risk factor related to the onset of symptomatic LBP.(76), (77) found robust evidence for the role of distress/depressive mood as a risk factor for chronic LBP, evidenced by a moderately large effect size (d=0.4).(78) Carroll et al(79) demonstrated that even after controlling for multiple potential confounding factors (demographic and socio-economic factors, health status, comorbidities, and previous spine-related injuries), individuals with the highest quartile of depression scores (ie, most depressed) were four times more likely to experience an onset of symptomatic neck or LBP within one year than individuals in the lowest quartile of scores. Despite the frequency of depression in the primary care setting and the availability of effective interventions, depression often goes undiagnosed.(80) Even if recognized, treatment often does not follow current treatment guidelines.((81) Therefore, biopsychosocial factors that play a significant role in identifying individuals at risk for the development of chronic pain will be considered. Specifically, we will examine the relevance of biopsychosocial factors by exploring the extent to which they represent a potential confounding variable in the development of chronic LBP. The following measures will be assessed:

Optimal Screening for Prediction of Referral and Outcome Yellow Flags assessment tool (OSPRO-YF) - Measures of psychosocial risk factors taken early after injury and sequentially over time represent an important

and relevant clinical necessity, as psychosocial risk factors can vary over time. Some preliminary work on how sequential assessment may improve predictive capabilities has been performed. In previous studies of the SBST, baseline risk status was compared to prediction from 4-week risk status, and 4-week change in risk status for prediction of 6 month pain and disability outcomes following low back pain(82). The results indicated that prediction improved when 4-week risk status or 4-week change in risk status was incorporated into predictive models. In particular, those with worsened 4 week change in psychosocial risk status (i.e. increasing pain associated distress) had notably worse 6 month outcomes. This provides promising preliminary data for investigating how these change patterns influence patient outcomes in patients with low back pain.

<u>Healthcare Utilization</u>
Finally, healthcare utilization data will be collected from the MHS Data Repository (MDR) database and will be confirmed via AHLTA. Healthcare utilization data will be used to determine any subsequent medical utilization related to low back pain. In order to collect this information a DUA will be completed between the researcher team and Patient Administration Systems and Biostatistics Activity (PASBA), and the Tricare Management Agency (TMA). Both of these agencies require a signed, completed IRB protocol prior to submitting the DUA. However, this should not impact the timing of this study; as the data pull will be completed no sooner than December 2016. This will provide more than enough time to complete the DUA with both agencies and submit the signed DUA with the BAMC IRB prior to performing this analysis. Details for determining the health care utilization are outlined below:

The goal of the MDR database will be to determine which of these subjects sought health care related to low back pain in the 12-month period prior to, as well as, the 12-month period after treatment in this study. This data (type, location, number of clinic visits, types of specialty clinic visits, imaging, and associated medication) will allow us to determine the extent of healthcare utilizations incidence.

- <u>Recording of Extracted Data with Identifiers:</u>
  The data will be provided in a coded manner from PASBA. As they have done before with members of our team on prior projects, they extract all the required data based upon the name, age and social security number that the Research Team provides to PASBA in order to identify the correct subjects that were in our study. PASBA will also assign a pseudo identification number matched with the list of our subject PHI that we provided to them. Therefore, the final working set they provide us for analysis will not have any identifying PHI/PII associated with it. If additional follow-up is needed to clarify a health care utilization event in AHLTA, the research team can check the master subject record (stored by the PI on an encrypted computer) in order to link the pseudo identification number to SSN. Therefore, prior to analysis occurring, only files with coded identifiers will be used. Confidentiality of protected health information will be maintained by the research staff at all times; however, it should be minimal at this time. The final working database to be used in the data analysis will not include PHI information.

- <u>Location of Extracted and Recorded Data:</u>
  The health care utilization data will be primarily extracted from the MDR database and through AHLTA. Even though the data is now coded, the extracted data will still be maintained in an encrypted, password protected file kept at the Physical Therapy Clinic, Brooke Army Medical Center by the PI. All data collection forms and the master participant list will be secured in an office at Brooke Army Medical Center in San Antonio, TX. All data maintained on a computer will be password protected and only accessible by the study investigators.

- Nature of Identifying Data
  Timeframes will be requested in reference to the baseline enrollment date rather than the actual date. For example, the date of appointment will be required initially to determine when the healthcare visit associated with the back pain occurred. However, this data will be coded differently in the working spreadsheet. The initial appointment will be recorded in weeks from the baseline examination. If multiple appointments exist, the range of dates will be recorded in the final spreadsheet (e.g. the patient was seen over a 4 week window) and the exact appointment days will not be included in the master spreadsheet. Analysis of the data will only occur in the coded spreadsheet.

- Status of the extracted data after completion of the research study:
  At the study completion, the file linking the participant to the study will be deleted. After a period of at least six years, the data will be destroyed in accordance with research department protocol. Full compliance with Health Insurance Portability and Accountability Act (HIPAA) standards will be upheld throughout the investigation to protect privacy and confidentiality.

- Redundancy:
  In addition to searching the MDR database, at each follow-up visit we will ask subjects if they have utilized healthcare resources since the last follow-up (4 and 26 weeks), specifically related to their LBP in 4 categories: visits to providers (traditional or complementary/alternative), medications (prescription or over-the-counter), interventions (injections, surgery, etc.), or testing (x-rays, MRI, etc.).

7.3.3. Human Biological Specimens/Tissue/Data Banking.
N/A

7.4 Statistical Consideration

7.4.1 Sample Size Estimation.
We will enroll 290 consecutive patients seen in primary care with a primary complaint of LBP who consent to participation. Patients will be enrolled in the primary care clinics at four military treatment facilities (MTFs). With a recruitment target of 290 subjects and a conservative recruitment rate of ~30 subjects per month (~10 per site), we will be able to complete recruitment within 1 year. Based on the fact that there are 6-8 new consultations for LBP per day in primary care, and given our previous experience, we do not anticipate having difficulty achieving our sample size within the study timeline.

Sample Size Estimation
The specific aims will be tested by use of pretreatment randomization allocation to low-risk, medium-risk, and high-risk groups in the intervention and control groups. The sample size calculation is based on the ability to detect a between group effect size of 0.3 at the 12-month primary endpoint. Based on a two-tailed significance level at an alpha level equal to 0.05, 80% power, and allowing for a 25% loss to follow-up, we will aim to recruit 290 participants, 145 in each group overall (Risk Stratified and Usual Care). Numbers below represent estimated recruitment targets at each site. However, one site may over-recruit in the case there are recruitment challenges at another site. Regardless, the total count between all 4 sites will not go over 290.

Estimate Required Sample Size	**234**
Estimate Participant Drop Out / Withdrawal	**56**

Total Enrollment Requirement	**290**

**Enrollment at Each Site**	
BAMC	**140**
MAMC	**50**
Womack	**50**
WBAMC	**50**

7.4.2 Primary (i.e., primary outcome variables) and secondary endpoints. The primary outcome variable is the changes in self-reported disability (Promis-29) between both groups at 1 year. The secondary endpoints are the other self-report variables (pain and quality of life), and the overall back-related healthcare utilization between both groups during the 1-year follow-up period.

7.4.3 Data Analysis
For the primary analyses, imputed datasets will be used for all descriptive and inferential assessments to address attrition bias, generated through multiple imputation (pooled estimates of five imputed datasets) by use of simulation based on a multivariate normal model (numerical variables) and a logistic regression model (categorical outcomes).(83) Estimates of treatment effect (mean difference for numerical outcomes, odds ratios for categorical outcomes, and incidence rate ratios for lost work days), with 95% CI, will be obtained using linear, binary logistic, ordinal logistic, and Poisson regression models, respectively, with adjustment for baseline score, age, sex, and back pain duration. Standardized effect sizes will be reported, and calculation of 95% CI for NNT according to the procedures described by Stang and colleagues.(84) Sensitivity analyses will be performed using complete-case analysis (ie, non-imputed dataset) and further adjustment for therapist's effects with random-effects modeling of the primary therapist. All analyses will be performed with SPSS (version 17.0.1).

The analysis of cost-effectiveness will be focused on the estimation of mean incremental quality-adjusted life years (QALYs) and back-pain-related health-care costs for the overall stratified management approach by use of a within-trial analysis. QALYs will be calculated with the EQ-5D. Details of the numbers of physical therapy sessions attended by each participant will be obtained through case report forms and an audit of clinical notes for the participating physical therapists. Other health care costs will be estimated from responses to the resource-use items contained within the 12 month self-report questionnaire. Similar to the clinical analysis, the economic evaluation will be replicated in the complete-case dataset. To assess the economic consequences of the stratified management intervention beyond healthcare resources, costs will also be assigned to self-reported work absence by use of the human capital approach. Self-reported work absence will be weighted by respondent-specific wage rates identified from data for yearly earnings and Standard Occupational Classification codes.(85) Because of the 12-month follow-up during the study, costs or health benefits will not be discounted. Analysis will be performed according to intention to treat principles.

7.7 Confidentiality.
Patients' confidentiality will be protected in the data collection process. All paper copies of study files will be stored in a locked cabinet, in a locked room at each study site. Consent forms that identify the patient by name will be stored in a locked cabinet separately from the remainder of the outcome instruments. These forms will also include the SSN for each subject. All research materials however, will be coded with unique patient identifiers (not the social security number) to protect subject identity, and patients will be instructed not to

identify themselves by name on any instrument. The data file linking the names and code numbers will be accessible only to the Principal Investigator, and data from each individual will be entered into a computer file by this code number. If the data are used in scholarly presentations or journal articles, the investigators will protect the anonymity of individual patients and will report only aggregate data (e.g. group means) where appropriate. No audio or videotaping of patients will be conducted as part of this study. The clinical care will also be documented in the patient's medical record according to usual practice. These methods for protection of patient's confidentiality have been required by the Institutional Review Boards and have been utilized by the researcher in previous studies. We therefore anticipate that these methods will be successful in protecting patients' confidentiality during this study.

No PHI/PII will be disclosed outside of DoD institutions. The local site PI at each non-SAMMC location will forward a list of subjects with linked PII to the primary investigator. This list will consist of the name and the SSN for each subject. This list is necessary for the data extraction from MDR. This document will be encrypted and password protected, and stored locally on the local site PI's encrypted computer until enrollment is complete. This file will then be sent via the U.S. Army Aviation and Missile Research Development and Engineering Center (AMRDC) Safe Access File Exchange system using both send and receive CAC encryption, or via encrypted .mil email. This is so that the overall study PI can send the list of PII to the data analyst for data extraction from MDR by the process we have previously outlined.

7.7.1 Certificate of Confidentiality. N/A

8.0 <u>RISKS/BENEFITS ASSESSMENT</u>

8.1 Risks.
Potential risks to subjects in this study are minimal. The procedures used in this study are standard procedures that are used in everyday clinical practice. The use of spinal manipulation for patients with LBP is supported by clinical practice guidelines in the United States and elsewhere. Manipulation of the lumbar spine is not associated with a high risk of serious side effects. The risks from the procedures used in this study are increased pain or muscle soreness as a result of the manipulation and exercise procedures used in physical therapy. Based on our clinical experience, the chances of this are unlikely, occurring in less than 25% of individuals. Most instances of increased pain or muscle soreness are transient, lasting less than 24 hours. We will attempt to minimize this risk by having licensed physical therapists specifically trained in the study procedures carry out all treatments. There is also a potential risk of psychological distress for the patient while answering self-report questions about the impact of the individual's LBP on various aspects of his or her life. Based on our clinical experience, the chance of this happening is rare, which means it occurs in less than 1% of people. To minimize this risk, patients will be told that they are not expected to answer any questions that are upsetting to them. There are no suicide screening questions on these forms. Those issues should be assessed as part of regular usual care when seeing the PCM, and are outside the scope of the proposed study. The questions included in this study are related to psychosocial risk factors associated with prognosis of musculoskeletal conditions (yellow flags) and have to do with catastrophizing, fear avoidance beliefs, etc. However, if the patient should mention any suicidal concerns in passing, during the enrollment process, their PCM will be notified and the patient will be escorted to see the nearest mental health care provider or emergency department, consistent with current clinical guidelines.

Although the risks to patients in this study will be generally low, we will implement monitoring procedures to ensure the safety and protection of subjects. All of the research team are credentialed providers, and familiar with managing patients with low back pain. The current primary care initiative in the Medical Homes is for

patient eventually to see a Physical Therapist directly for low back pain, instead of seeing a PCM, so this aligns well with that initiative and will soon be usual care. In the Moreno Clinic, this is not yet the case at the time of beginning the study. The research team will always monitor the safety and appropriateness of each patient in the study. We have discussed mental health issue awareness in the previous section. We will also screen for all red flags (appendix ?? - red flag questionnaire), in addition ot the screening performed by the patients PCM. All personnel involved with the research who will be responsible for collecting and handling the data will have completed the CITI training and will be trained by one of the investigators. Protection of patient confidentiality and procedures for reporting adverse events will be included in these training sessions. Any adverse events occurring as a result of participation in this study will be reported immediately to the principal investigator and the Institutional Review Board involved with the study. The Principal Investigator will meet on a weekly basis with the study staff to review study progress, including any adverse events or breeches in patient confidentiality.

8.2 Potential Benefits.
Chronic disability related to LBP within the MHS imposes a tremendous economic burden to the health care system and a burdensome amount of pain and suffering to afflicted individuals. Subjects in this study will benefit from the use of an active management approach, including reassurance and advice to remain active, which has been shown to be an effective management strategy for patients with LBP. Both groups will receive the active management approach. It is unknown at this time whether there will be a greater benefit gained by the addition of the Risk-Stratified Care. The results of this study will also benefit the rehabilitation and primary care medicine communities by adding to the current literature on the most effective management strategies for subgroups of patients with LBP managed in primary care. This study would be the first clinical trial to attempt to implement a risk-stratification procedure within primary care in the MHS to direct patients to the management approach most likely to benefit them. The results would also provide evidence on the cost-effectiveness of such an approach. Given the low level of potential risks involved in this study, the potential benefits would appear to outweigh the potential risks to subjects.

Subjects participating in this study will have at least as good a chance of controlling low back pain with other types of treatments. For example, they may benefit from the education and advice, which has been shown to be an effective management strategy for patients with low back pain. It is unknown at this time whether there will be a greater benefit gained by individuals who receive additional physical therapy visits. Regardless of which group a subject is in, there is no guarantee or promise of benefit from this study.

This study may also benefit others by helping to find out whether the treatments used in this study are better than other approaches for managing low back pain. The results will also help better understand the cost-effectiveness of treatments in this study.

9.0 ADVERSE EVENTS, UNANTICIPATED PROBLEMS, AND DEVIATIONS

9.1 All procedures utilized are used within standard of care settings. No adverse events are anticipated related to this study. Any adverse events that do occur will be treated in the same manner as if they occurred with patients receiving treatment outside of the study. Expected adverse events related to treatment in this study, which are not serious will be reported on the Annual Progress Report (APR) during the continuing review of the protocol.

Deviations:

Minor protocol deviations will be reported to the IRB during annual review using the protocol deviation tracking log P53. Major deviations will be reported to the IRB within 48 hours by the primary investigator, in accordance with HRPP policy memo 5.4 on protocol deviations.

9.2 Reporting Unanticipated Problems Involving Risks to Subjects or Others, Serious Adverse Events and Deaths to the Office of the IRB, BAMC.

All unanticipated problems involving risk to subjects or others, serious adverse events, and all subject deaths related to the study will be reported within 48 hours of the research team's knowledge of the event by phone (210-916-0606), by e-mail (BAMC_IRB_AE@amedd.army.mil), by facsimile (210-916-1650) or via letter addressed to Human Protections Administrator, Office of the Institutional Review Board, Brooke Army Medical Center, Attn: MCHE-CI,3698 Chambers Pass, Fort Sam Houston, TX 78234-6315. A complete written report will follow the initial notification within 10 working days.

9.3 Research Monitor. *N/A*

10.0 WITHDRAWAL FROM STUDY PARTICIPATION. Subjects may withdraw at any time by just notifying a member of the investigative team. The secondary endpoint is healthcare utilization being collected through the MDR database, and therefore it will not be affected with patient withdrawal. However, self-report data will not be collected at the 1, 6, and 12-month follow-up points if patients choose to withdraw and not make those follow-up appointments.

11.0 USAMRMC Volunteer Registry Database. N/A

12.0 REFERENCES.

1. Cohen SP, Brown C, Kurihara C, Plunkett A, Nguyen C. Diagnoses and factors associated with medical evacuation and return to duty for service members participating in Operation Iraqi Freedom or Operation Enduring …. Lancet [Internet]. Elsevier; 2010; Available from: http://www.sciencedirect.com/science/article/pii/S0140673609617979

2. Cohen SP, Nguyen C, Kapoor SG, Anderson-Barnes VC, Foster L, Shields C, et al. Back pain during war: an analysis of factors affecting outcome. Arch Intern Med. 2009 Nov 9;169(20):1916–23.

3. Armed Forces Health Surveillance Center (AFHSC). Ambulatory visits among members of the active component, U.S. Armed Forces, 2010. MSMR. 2011 Apr;18(4):16–21.

4. MSMR. Low back pain, active component, U.S. Armed Forces 2000-2009. Medical Surveillance Monthly Report. Aberdeen Proving Ground, MD; 2010;

5. Knox J, Orchowski J, Scher DL, Owens BD, Burks R, Belmont PJ. The incidence of low back pain in active duty United States military service members. Spine . 2011 Aug 15;36(18):1492–500.

6. Cohen SP, Griffith S, Larkin TM, Villena F, Larkin R. Presentation, diagnoses, mechanisms of injury, and treatment of soldiers injured in Operation Iraqi Freedom: an epidemiological study conducted at two military pain management centers. Anesth Analg. 2005 Oct;101(4):1098–103, table of contents.

7. Lincoln AE, Smith GS, Amoroso PJ, Bell NS. The natural history and risk factors of musculoskeletal conditions resulting in disability among US Army personnel. Work. 2002;18(2):99–113.

8. Morasco BJ, Duckart JP, Carr TP, Deyo RA, Dobscha SK. Clinical characteristics of veterans prescribed high doses of opioid medications for chronic non-cancer pain. Pain. 2010 Dec;151(3):625–32.

9. Deyo RA, Phillips WR. Low back pain. A primary care challenge. Spine . 1996 Dec 15;21(24):2826–32.

10. Gellhorn AC, Chan L, Martin B, Friedly J. Management Patterns in Acute Low Back Pain. Spine [Internet]. 2010; Available from: http://www.researchgate.net/profile/Janna_Friedly/publication/49630728_Management_patterns_in_acute_low_back_pain_the_role_of_physical_therapy/links/09e415110525f9a701000000.pdf

11. Hart LG, Deyo RA, Cherkin DC. Physician office visits for low back pain. Frequency, clinical evaluation, and treatment patterns from a U.S. national survey. Spine . 1995 Jan 1;20(1):11–9.

12. Feuerstein M, Hartzell M, Rogers HL, Marcus SC. Evidence-based practice for acute low back pain in primary care: patient outcomes and cost of care. Pain. 2006 Sep;124(1-2):140–9.

13. Panel E. Veteran's Health Administration/Department of Defense Clinical Practice Guideline for the Management of Low Back Pain or Sciatica in the Primary Care Setting. The Office of Performance and Quality, Veteran's Health Administration and the Quality Management Directorate, United States Army Medical Command; 1999.

14. Chou R, Huffman LH, American Pain Society, American College of Physicians. Nonpharmacologic therapies for acute and chronic low back pain: a review of the evidence for an American Pain Society/American College of Physicians clinical practice guideline. Ann Intern Med. 2007 Oct 2;147(7):492–504.

15. Lamb SE, Lall R, Hansen Z, Castelnuovo E, Withers EJ, Nichols V, et al. A multicentred randomised controlled trial of a primary care-based cognitive behavioural programme for low back pain. The Back Skills Training (BeST) trial. Health Technol Assess. 2010 Aug;14(41):1–253, iii – iv.

16. UK BEAM Trial Team. United Kingdom back pain exercise and manipulation (UK BEAM) randomised trial: effectiveness of physical treatments for back pain in primary care. BMJ. 2004 Dec 11;329(7479):1377.

17. Low Back Pain: Early Management of Persistent Non-specific Low Back Pain. London: Royal College of General Practitioners (UK); 2010.

18. Nelson CF, Metz RD, LaBrot T. Effects of a managed chiropractic benefit on the use of specific diagnostic and therapeutic procedures in the treatment of low back and neck pain. J Manipulative Physiol Ther. 2005 Oct;28(8):564–9.

19. Luo X, Pietrobon R, Hey L. Patterns and trends in opioid use among individuals with back pain in the United States. Spine . 2004 Apr 15;29(8):884–90; discussion 891.

20. Deyo RA, Mirza SK, Turner JA, Martin BI. Overtreating chronic back pain: time to back off? J Am Board Fam Med. 2009 Jan;22(1):62–8.

21. Friedly J, Chan L, Deyo R. Increases in lumbosacral injections in the Medicare population: 1994 to 2001. Spine . 2007 Jul 15;32(16):1754–60.

22. Weiner DK, Kim Y-S, Bonino P, Wang T. Low back pain in older adults: are we utilizing healthcare resources wisely? Pain Med. 2006 Mar;7(2):143–50.

23. Deyo RA, Mirza SK. Trends and variations in the use of spine surgery. Clin Orthop Relat Res. 2006 Feb;443:139–46.

24. Gray DT, Deyo RA, Kreuter W, Mirza SK, Heagerty PJ, Comstock BA, et al. Population-based trends in volumes and rates of ambulatory lumbar spine surgery. Spine . 2006 Aug 1;31(17):1957–63; discussion 1964.

25. Williams CM, Maher CG, Hancock MJ, McAuley JH, McLachlan AJ, Britt H, et al. Low back pain and best practice care: A survey of general practice physicians. Arch Intern Med. 2010 Feb 8;170(3):271–7.

26. Freburger JK, Holmes GM, Agans RP, Jackman AM, Darter JD, Wallace AS, et al. The rising prevalence of chronic low back pain. Arch Intern Med. 2009 Feb 9;169(3):251–8.

27. Martin BI, Deyo RA, Mirza SK, Turner JA, Comstock BA, Hollingworth W, et al. Expenditures and health status among adults with back and neck problems. JAMA. 2008 Feb 13;299(6):656–64.

28. Foster NE, Hill JC, Hay EM. Subgrouping patients with low back pain in primary care: are we getting any better at it? Man Ther. 2011 Feb;16(1):3–8.

29. Van der Windt D, Hay E, Jellema P, Main C. Psychosocial interventions for low back pain in primary care: lessons learned from recent trials. Spine . 2008 Jan 1;33(1):81–9.

30. McGrail MP Jr, Lohman W, Gorman R. Disability prevention principles in the primary care office. Am Fam Physician. 2001 Feb 15;63(4):679–84.

31. Imison C, Naylor C. Referral management: lessons for success. London: King’s Fund, 2010.

32. Koes BW, van Tulder M, Lin C-WC, Macedo LG, McAuley J, Maher C. An updated overview of clinical guidelines for the management of non-specific low back pain in primary care. Eur Spine J. 2010 Dec;19(12):2075–94.

33. Murphy AYMT, van Teijlingen ER, Gobbi MO. Inconsistent grading of evidence across countries: a review of low back pain guidelines. J Manipulative Physiol Ther. 2006 Sep;29(7):576–81, 581.e1–2.

34. Atlas SJ, Deyo RA. Evaluating and managing acute low back pain in the primary care setting. J Gen Intern Med. 2001 Feb;16(2):120–31.

35. Korff M Von, Moore JC. Stepped care for back pain: activating approaches for primary care. Ann Intern Med. 2001 May 1;134(9 Pt 2):911–7.

36. Korff M Von, Saunders K. The course of back pain in primary care. Spine . 1996 Dec 15;21(24):2833–7; discussion 2838–9.

37. Dunn KM, Jordan K, Croft PR. Characterizing the course of low back pain: a latent class analysis. Am J Epidemiol. 2006 Apr 15;163(8):754–61.

38. Hill JC, Whitehurst DGT, Lewis M, Bryan S, Dunn KM, Foster NE, et al. Comparison of stratified primary care management for low back pain with current best practice (STarT Back): a randomised controlled trial. Lancet. 2011 Oct 29;378(9802):1560–71.

39. Chou R, Qaseem A, Owens DK, Shekelle P, Clinical Guidelines Committee of the American College of Physicians. Diagnostic imaging for low back pain: advice for high-value health care from the American College of Physicians. Ann Intern Med. 2011 Feb 1;154(3):181–9.

40. Chou R, Huffman LH, American Pain Society, American College of Physicians. Medications for acute and chronic low back pain: a review of the evidence for an American Pain Society/American College of Physicians clinical

practice guideline. Ann Intern Med. 2007 Oct 2;147(7):505–14.

41. Fritz JM, Cleland JA, Childs JD. Subgrouping patients with low back pain: evolution of a classification approach to physical therapy. J Orthop Sports Phys Ther. 2007 Jun;37(6):290–302.

42. Rhon D, Fritz J. COMParative Early Treatment Effectiveness between physical therapy and usual care for low back pain (COMPETE): study protocol for a randomized controlled trial. Trials. 2015 Sep 23;16(1):423.

43. Klaber Moffett J, Main C, Roland M, Waddell G. The back book. The Stationary Office Books. 2002;

44. Burton AK, Waddell G, Tillotson KM, Summerton N. Information and advice to patients with back pain can have a positive effect. A randomized controlled trial of a novel educational booklet in primary care. Spine . 1999 Dec 1;24(23):2484–91.

45. Coudeyre E, Tubach F, Rannou F, Baron G, Coriat F, Brin S, et al. Effect of a simple information booklet on pain persistence after an acute episode of low back pain: a non-randomized trial in a primary care setting. PLoS One. 2007 Aug 8;2(8):e706.

46. Vlaeyen JW, de Jong J, Geilen M, Heuts PH, van Breukelen G. Graded exposure in vivo in the treatment of pain-related fear: a replicated single-case experimental design in four patients with chronic low back pain. Behav Res Ther. 2001 Feb;39(2):151–66.

47. Thomas E, Croft PR, Paterson SM, Dziedzic K, Hay EM. What influences participants' treatment preference and can it influence outcome? Results from a primary care-based randomised trial for shoulder pain. Br J Gen Pract. 2004 Feb;54(499):93–6.

48. Fritz JM, Childs JD, Flynn TW. Pragmatic application of a clinical prediction rule in primary care to identify patients with low back pain with a good prognosis following a brief spinal manipulation intervention. BMC Fam Pract. 2005 Jul 14;6(1):29.

49. Linton SJ, Andersson T. Can chronic disability be prevented? A randomized trial of a cognitive-behavior intervention and two forms of information for patients with spinal pain. Spine . 2000 Nov 1;25(21):2825–31; discussion 2824.

50. McCracken LM, Turk DC. Behavioral and Cognitive–Behavioral Treatment for Chronic Pain: Outcome, Predictors of Outcome, and Treatment Process. Spine . 2002 Nov 15;27(22):2564.

51. Browder DA, Childs JD, Cleland JA, Fritz JM. Effectiveness of an extension-oriented treatment approach in a subgroup of subjects with low back pain: a randomized clinical trial. Phys Ther. 2007 Dec;87(12):1608–18; discussion 1577–9.

52. Choi BKL, Verbeek JH, Tam WW-S, Jiang JY. Exercises for prevention of recurrences of low-back pain. Occup Environ Med. 2010 Nov;67(11):795–6.

53. Macedo LG, Maher CG, Latimer J, McAuley JH. Motor control exercise for persistent, nonspecific low back pain: a systematic review. Phys Ther. 2009 Jan;89(1):9–25.

54. Staal JB, Hlobil H, Köke AJA, Twisk JWR, Smid T, van Mechelen W. Graded activity for workers with low back pain: who benefits most and how does it work? Arthritis Rheum. 2008 May 15;59(5):642–9.

55. Lambeek LC, Bosmans JE, Van Royen BJ, Van Tulder MW, Van Mechelen W, Anema JR. Effect of integrated care for sick listed patients with chronic low back pain: economic evaluation alongside a randomised controlled trial. BMJ. 2010 Nov 30;341:c6414.

56. Vlaeyen JW, Linton SJ. Fear-avoidance and its consequences in chronic musculoskeletal pain: a state of the art. Pain. 2000 Apr;85(3):317–32.

57. Lindström I, Ohlund C, Eek C, Wallin L, Peterson LE, Nachemson A. Mobility, strength, and fitness after a graded activity program for patients with subacute low back pain. A randomized prospective clinical study with a behavioral therapy approach. Spine . 1992 Jun;17(6):641–52.

58. Hill JC, Dunn KM, Lewis M, Mullis R, Main CJ, Foster NE, et al. A primary care back pain screening tool: identifying patient subgroups for initial treatment. Arthritis Rheum. 2008 May 15;59(5):632–41.

59. Hill JC, Vohora K, Dunn KM, Main CJ, Hay EM. Comparing the STarT back screening tool's subgroup allocation of individual patients with that of independent clinical experts. Clin J Pain. 2010 Nov;26(9):783–7.

60. Hill JC, Dunn KM, Main CJ, Hay EM. Subgrouping low back pain: a comparison of the STarT Back Tool with the Orebro Musculoskeletal Pain Screening Questionnaire. Eur J Pain. 2010 Jan;14(1):83–9.

61. Hay EM, Dunn KM, Hill JC, Lewis M, Mason EE, Konstantinou K, et al. A randomised clinical trial of subgrouping and targeted treatment for low back pain compared with best current care. The STarT Back Trial Study Protocol. BMC Musculoskelet Disord. 2008 Apr 22;9:58.

62. Devilly GJ, Borkovec TD. Psychometric properties of the credibility/expectancy questionnaire. J Behav Ther Exp Psychiatry. 2000 Jun;31(2):73–86.

63. Smeets RJEM, Beelen S, Goossens MEJB, Schouten EGW, Knottnerus JA, Vlaeyen JWS. Treatment expectancy and credibility are associated with the outcome of both physical and cognitive-behavioral treatment in chronic low back pain. Clin J Pain. 2008 May;24(4):305–15.

64. Roland M, Morris R. A study of the natural history of back pain. Part I: development of a reliable and sensitive measure of disability in low-back pain. Spine . 1983 Mar;8(2):141–4.

65. Deyo RA, Battie M, Beurskens AJ, Bombardier C, Croft P, Koes B, et al. Outcome measures for low back pain research. A proposal for standardized use. Spine . 1998 Sep 15;23(18):2003–13.

66. Jctdj F, Couper J, O'Brien JP. The Oswestry low back pain disability questionaire. Physiotherapy. 1980;66:271–3.

67. Fairbank JC, Pynsent PB. The Oswestry Disability Index. Spine . 2000 Nov 15;25(22):2940–52; discussion 2952.

68. Fritz JM, Irrgang JJ. A comparison of a modified Oswestry Low Back Pain Disability Questionnaire and the Quebec Back Pain Disability Scale. Phys Ther. 2001 Feb;81(2):776–88.

69. PROMIS [Internet]. [cited 2015 Nov 12]. Available from: http://www.nihpromis.org/measures/availableinstruments.

70. Cella D, Riley W, Stone A, Rothrock N, Reeve B, Yount S, et al. The Patient-Reported Outcomes Measurement Information System (PROMIS) developed and tested its first wave of adult self-reported health outcome item banks: 2005-2008. J Clin Epidemiol. 2010 Nov;63(11):1179–94.

71. Xia T, Wilder DG, Gudavalli MR, DeVocht JW, Vining RD, Pohlman KA, et al. Study protocol for patient response to spinal manipulation - a prospective observational clinical trial on physiological and patient-centered outcomes in patients with chronic low back pain. BMC Complement Altern Med. 2014 Aug 8;14:292.

72. Chan CW, Goldman S, Ilstrup DM, Kunselman AR, O'Neill PI. The pain drawing and Waddell's nonorganic physical signs in chronic low-back pain. Spine . 1993 Oct 1;18(13):1717–22.

73. EuroQol Group. EuroQol--a new facility for the measurement of health-related quality of life. Health Policy. 1990 Dec;16(3):199–208.

74. Sullivan PW, Ghushchyan V. Preference-Based EQ-5D index scores for chronic conditions in the United States. Med Decis Making. 2006 Jul;26(4):410–20.

75. Dolan P, Gudex C, Kind P, Williams A. The time trade-off method: results from a general population study. Health Econ. 1996 Mar;5(2):141–54.

76. Klenerman L, Slade PD, Stanley IM, Pennie B, Reilly JP, Atchison LE, et al. The prediction of chronicity in patients with an acute attack of low back pain in a general practice setting. Spine . 1995 Feb 15;20(4):478–84.

77. Pincus T, Vlaeyen JWS, Kendall NAS, Korff MR Von, Kalauokalani DA, Reis S. Cognitive-behavioral therapy and psychosocial factors in low back pain: directions for the future. Spine . 2002 Mar 1;27(5):E133–8.

78. Pincus T, Burton AK, Vogel S, Field AP. A systematic review of psychological factors as predictors of chronicity/disability in prospective cohorts of low back pain. Spine . 2002 Mar 1;27(5):E109–20.

79. Carroll LJ, Cassidy JD, Côté P. Depression as a risk factor for onset of an episode of troublesome neck and low back pain. Pain. 2004 Jan;107(1-2):134–9.

80. Waddell G, Newton M, Henderson I, Somerville D, Main CJ. A Fear-Avoidance Beliefs Questionnaire (FABQ) and the role of fear-avoidance beliefs in chronic low back pain and disability. Pain. 1993 Feb;52(2):157–68.

81. Gilbody SM, House AO, Sheldon TA. Routinely administered questionnaires for depression and anxiety: systematic review. BMJ. 2001 Feb 17;322(7283):406–9.

82. Beneciuk JM, Fritz JM, George SZ. The STarT Back Screening Tool for prediction of 6-month clinical outcomes: relevance of change patterns in outpatient physical therapy settings. J Orthop Sports Phys Ther. 2014 Sep;44(9):656–64.

83. Little RJA, Rubin DB. Statistical analysis with missing data. John Wiley & Sons; 2014.

84. Stang A, Poole C, Bender R. Common problems related to the use of number needed to treat. J Clin Epidemiol. 2010 Aug;63(8):820–5.

85. Pike R. Patterns of pay: Results of the Annual Survey of Hours and Earnings 1997 to 2010. Economic and Labour Market Review. Nature Publishing Group; 2011 Mar 1;5(3):14–40.

13.0 TIME REQUIRED TO COMPLETE THE RESEARCH (including data analysis).

With an anticipated low back pain population of approximately 40-50 patients per month and a conservative enrollment rate of 50%, we anticipate 1 year for the enrollment period.

- November 2015 – Protocol submitted to BAMC IRB
- February 2016 – Anticipate IRB approval
- March - May 2016 – Study staff training on study procedures
- June 2016 – Subject recruitment/enrollment begins
- June 2017 – Subject recruitment/enrollment end
- June 2018 – Last subject completes 1-year follow-up
- August 2018 – Healthcare utilization data requested from PASBA
- December 2018 – Data analysis and sub-analysis complete

- February 2019 – Publication submitted to appropriate journal

14.0 STUDY CLOSURE PROCEDURES

In accordance to the Human Research Protection Program Policy Memorandum 5.5, a protocol closure report, Form P35, will be submitted following completion of all data analysis and manuscript has been submitted. The closure report will also include summary of subject enrollment, a summary of any unreported issues, adverse events, and publications and presentations planned. Data will also be deidentified as previously mentioned and retained for future research on a secure encrypted server. The ICD will be kept for 3 years and the HIPAA for 6 yrs in a locked file cabinet in the locked office of the Chief of Physical Therapy at the CFI.
