# Supplementary material for: Effect of Risk-Stratified Care on Disability Among Adults With Low Back Pain Treated in the Military Health System: A Randomized Clinical Trial
Source: JAMA Netw Open. 2023 Jul 6;6(7):e2321929. doi: 10.1001/jamanetworkopen.2023.21929 (PMC10326636; doi:10.1001/jamanetworkopen.2023.21929)
Supplement: Supplement 2. — eFigure 1. Roland Morris Disability Questionnaire (RMDQ): Between Groups Over Time (LS Means) eFigure 2. RMDQ Between-Group Comparison (Observed Values, Raw) eFigure 3. PROMIS Pain Interference Between-Groups Over Time (LS Means) eFigure 4. PROMIS Physical Function Between-Groups Over Time (LS Means) eTable 1. Demographic Differences for Individuals With Only Baseline Data vs Individuals With at Least 1 Follow-up ( mITT Population) eTable 2. Number of Low Back Pain (LBP)–Related Visits and Costs By Risk Strata eTable 3. Number of Low Back Pain (LBP)–Related Visits by Risk Strata and Treatment eTable 4. Summary of OSPRO-Yellow Flag Count by Treatment Group (Observed Values) eTable 5. Mean RMDQ Scores at Each Time Point by Treatment Group (Observed Values) eTable 6. Mean PROMIS Pain Interference Scores at Each Time Point by Treatment Group (Observed Values) eTable 7. Mean PROMIS Physical Function Scores at Each Time Point by Treatment Group (Observed Values) eTable 8. Mean PROMIS Sleep Disturbance Scores at Each Time Point by Treatment Group (Observed Values) eTable 9. COVID-19 Impact by Treatment Group eTable 10. Usual Care Intervention Recommended Reporting Elements eReferences. [file jamanetwopen-e2321929-s002.pdf]

Supplemental Online Content

Rhon DI, Greenlee TA, Poehlein E, et al. Effect of risk-stratified care on disability among adults with low back pain being treated at the Military Health System: a randomized clinical trial. *JAMA Netw Open.* 2023;6(7):e2321929. doi:10.1001/jamanetworkopen.2023.21929

eFigure 1. Roland Morris Disability Questionnaire (RMDQ): Between Groups Over Time (LS Means)

eFigure 2. RMDQ Between-Group Comparison (Observed Values, Raw)

eFigure 3. PROMIS Pain Interference Between-Groups Over Time (LS Means)

eFigure 4. PROMIS Physical Function Between-Groups Over Time (LS Means)

eTable 1. Demographic Differences for Individuals With Only Baseline Data vs Individuals With at Least 1 Follow-up (mITT Population)

eTable 2. Number of Low Back Pain (LBP)–Related Visits and Costs By Risk Strata

eTable 3. Number of Low Back Pain (LBP)–Related Visits by Risk Strata and Treatment

eTable 4. Summary of OSPRO-Yellow Flag Count by Treatment Group (Observed Values)

eTable 5. Mean RMDQ Scores at Each Time Point by Treatment Group (Observed Values)

eTable 6. Mean PROMIS Pain Interference Scores at Each Time Point by Treatment Group (Observed Values)

eTable 7. Mean PROMIS Physical Function Scores at Each Time Point by Treatment Group (Observed Values)

eTable 8. Mean PROMIS Sleep Disturbance Scores at Each Time Point by Treatment Group (Observed Values)

eTable 9. COVID-19 Impact by Treatment Group

eTable 10. Usual Care Intervention Recommended Reporting Elements

eReferences.

This supplemental material has been provided by the authors to give readers additional information about their work.

eFigure 1. Roland Morris Disability Questionnaire (RMDQ): Between Groups Over Time (LS Means)

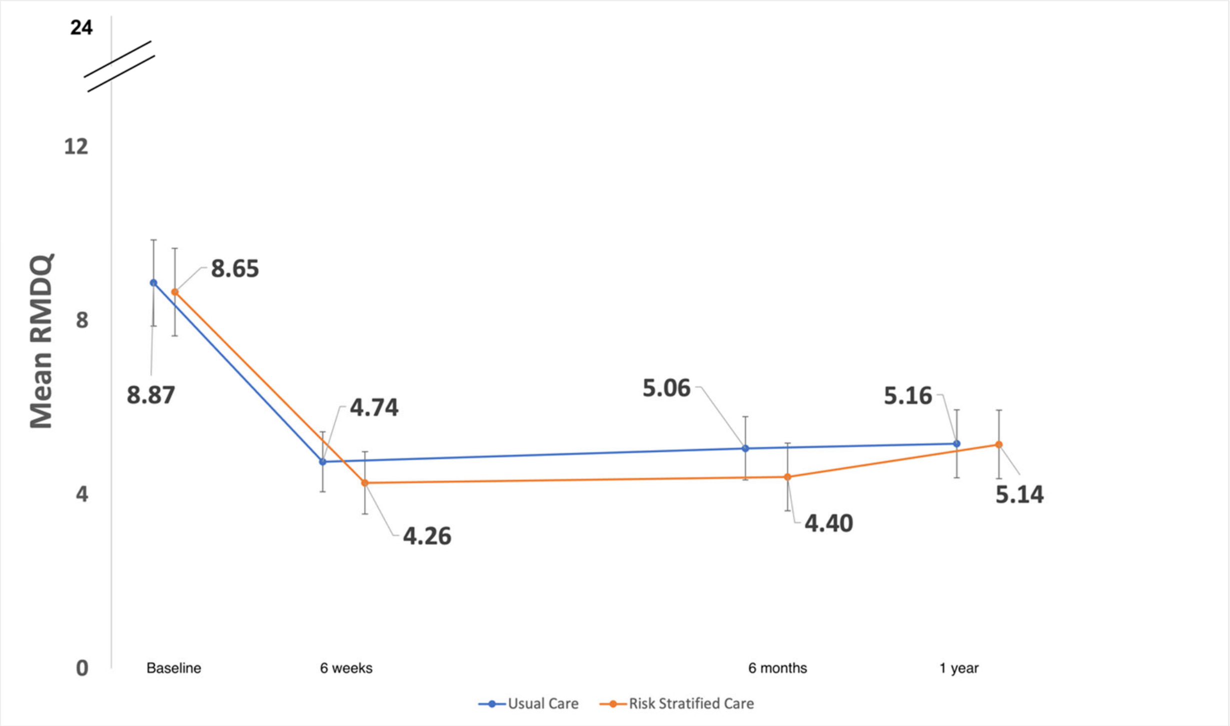

© 2023 Rhon DI et al. *JAMA Network Open.*

eFigure 2. RMDQ Between-Group Comparison (Observed Values, Raw)

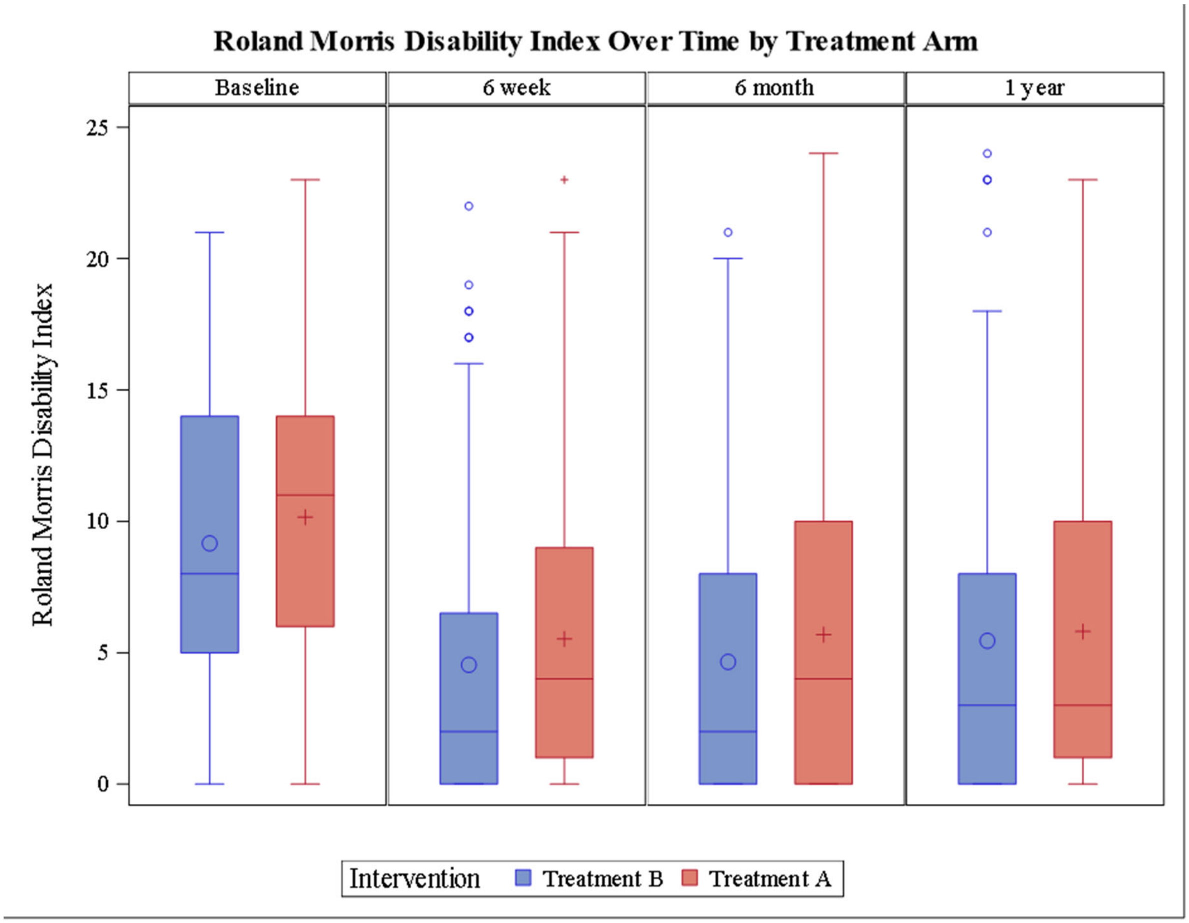

Figure key: Treatment A = Usual Care and Treatment B = Risk-Stratified Care. Horizontal line within the box indicates the median, while the top and bottom of the box indicate the 25th and 75th quartiles. O and + indicate the mean values in the risk-stratified and usual care groups, respectively.

© 2023 Rhon DI et al. *JAMA Network Open.*

eFigure 3. PROMIS Pain Interference Between-Groups Over Time (LS Means)

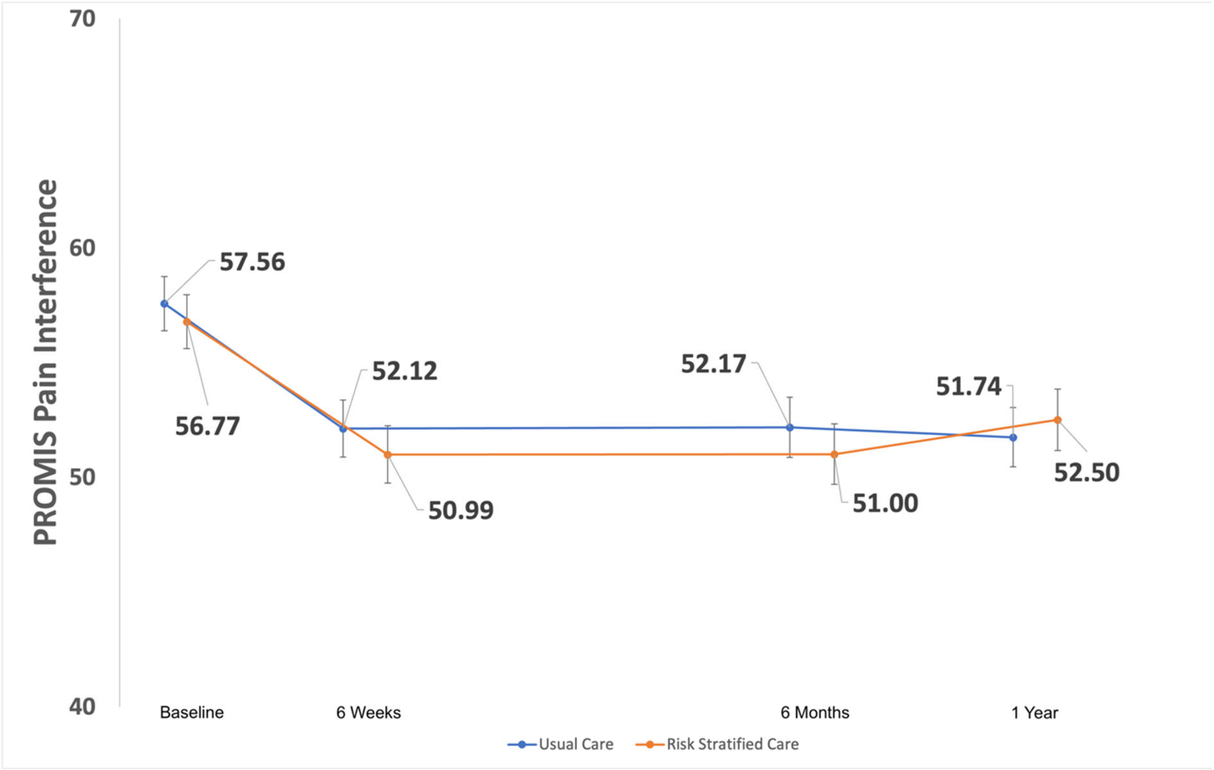

© 2023 Rhon DI et al. *JAMA Network Open.*

eFigure 4. PROMIS Physical Function Between-Groups Over Time (LS Means)

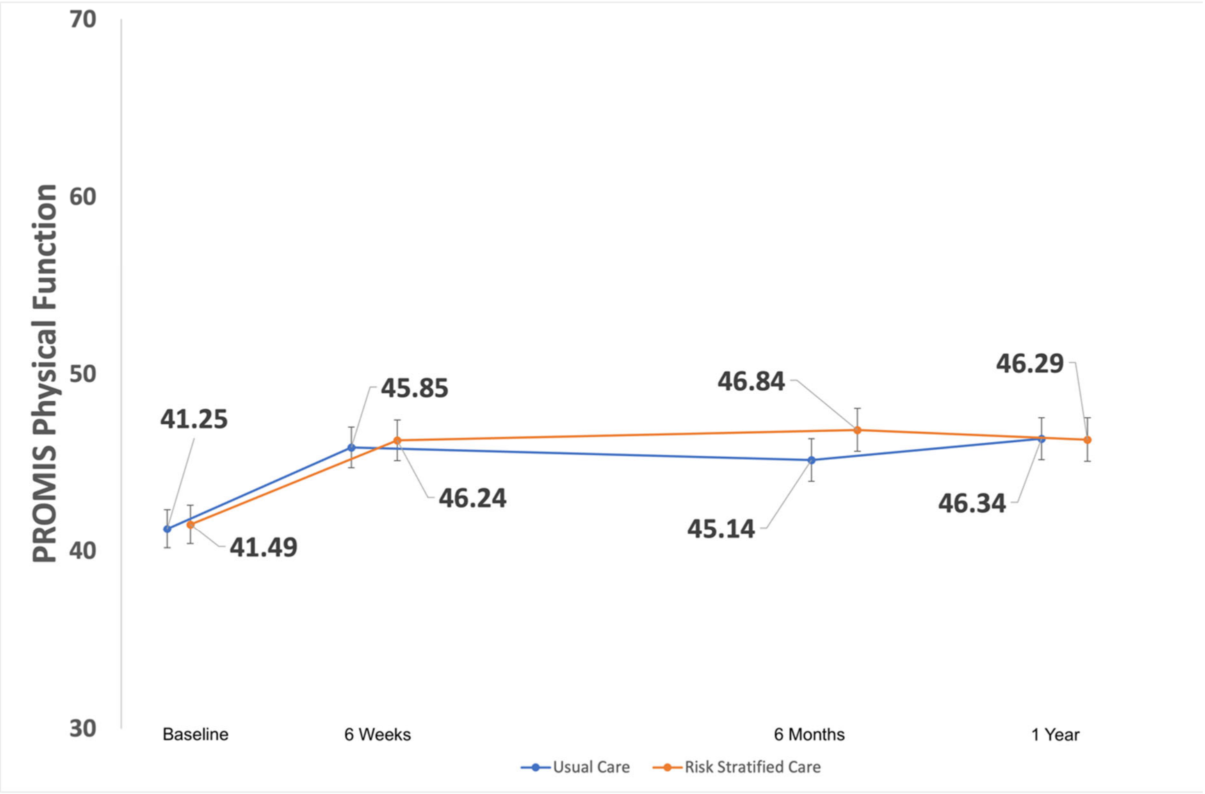

© 2023 Rhon DI et al. *JAMA Network Open.*

eTable 1. Demographic Differences for Individuals With Only Baseline Data vs Individuals With at Least 1 Follow-up (mITT Population)

	Population, N (%)	
	Only Baseline RMDQ (N=20)	RMDQ at Baseline and Follow-up (N=270)
Age (Years), mean (SD)	32.0 (8.6)	34.3 (8.4)
Median (Q1, Q3)	32.0 (24.5, 37.5)	35.0 (27.0, 40.0)
Min-Max	(19.0-48.0)	(19.0-50.0)
Female Sex	8 (40.0%)	91 (33.7%)
Race		
- Native American*	0 (0.0%)	5 (1.9%)
- Asian	1 (5.0%)	17 (6.3%)
- Black or African American	5 (25.0%)	53 (19.6%)
- White	9 (45.0%)	148 (54.8%)
- More than one race	2 (10.0%)	20 (7.4%)
- Other	3 (15.0%)	27 (10.0%)
Hispanic/Latino Ethnicity	7 (35.0%)	79 (29.3%)
Intervention		
- Usual Care	11 (55.0%)	134 (49.6%)
- Risk-Stratified Care	9 (45.0%)	136 (50.4%)

mITT = Modified Intention to Treat; Q = quartile; RMDQ = Roland Morris Disability Questionnaire;
* includes American Indian, Alaskan Native, or Native Hawaiian/Pacific Islander

© 2023 Rhon DI et al. *JAMA Network Open.*

eTable 2. Number of Low Back Pain (LBP)–Related Visits and Costs By Risk Strata

	Low Risk (N=160)	Medium Risk (N=109)	High Risk (N=21)
**Number of LBP visits for any reason, 1-year follow-up**			
Mean (SD)	5.8 (6.7)	8.5 (9.9)	14.6 (9.4)
Median (Q1, Q3)	3.0 (1.0-8.0)	5.0 (2.0-10.0)	13.0 (7.0, 20.0)
Min-Max	(0.0-43.0)	(0.0-48.0)	(1.0-34.0)
**Number of LBP visits for any reason within first 90 days**			
Mean (SD)	2.8 (3.3)	4.9 (4.9)	6.4 (4.1)
Median (Q1, Q3)	2.0 (1.0, 3.0)	4.0 (2.0, 7.0)	6.0 (4.0, 9.0)
Min-Max	(0.0-26.0)	(0.0-35.0)	(0.0-17.0)
**Number of LBP Physical Therapy Visits, 1-year follow-up**			
Mean (SD)	2.4 (3.5)	3.4 (4.1)	4.0 (3.4)
Median (Q1, Q3)	1.0 (1.0, 2.0)	2.0 (1.0, 5.0)	4.0 (1.0, 5.0)
Min-Max	(0.0-21.0)	(0.0-21.0)	(0.0-11.0)
**Number of LBP Physical Therapy Visits within first 90 days**			
Mean (SD)	1.4 (1.8)	2.7 (3.3)	2.8 (2.8)
Median (Q1, Q3)	1.0 (1.0, 1.0)	2.0 (1.0, 3.0)	2.0 (1.0, 4.0)
Min-Max	(0.0-12.0)	(0.0-17.0)	(0.0-11.0)

SD = standard deviation; Q = quartile

In the Usual Care group, 88 participants (60.7%) had physical therapy that started within 90 days from enrollment (N = 83 that had initiated physical therapy within 60 days from enrollment).

eTable 3. Number of Low Back Pain (LBP)–Related Visits by Risk Strata and Treatment

	Risk Stratified Care (N=145)			Usual care† (N = 145)
	Low Risk, Stratified (N=83)	Medium Risk, Stratified (N=52)	High Risk, Stratified (N=10)	
**Number of LBP visits for any reason, 1-year follow-up**				
Mean (SD)	6.0 (6.1)	9.2 (10.3)	14.3 (9.1)	7.1 (8.8)
Median (Q1, Q3)	4.0 (2.0, 9.0)	6.0 (3.5, 9.5)	12.5 (7.0, 20.0)	3.0 (1.0, 11.0)
Min-Max	(1.0-38.0)	(1.0-45.0)	(4.0-34.0)	(0.0-48.0)
**Number of LBP visits for any reason within first 90 days**				
Mean (SD)	2.9 (2.4)	6.0 (5.3)	6.4 (3.6)	3.4 (4.2)
Median (Q1, Q3)	2.0 (1.0, 4.0)	4.0 (3.0, 8.0)	5.5 (4.0, 9.0)	2.0 (1.0, 4.0)
Min-Max	(1.0-12.0)	(1.0-35.0)	(2.0-13.0)	(0.0-26.0)
**Number of LBP Physical Therapy Visits, 1-year follow-up**				
Mean (SD)	2.4 (3.7)	4.5 (4.0)	4.6 (3.0)	2.4 (3.5)
Median (Q1, Q3)	1.0 (1.0, 2.0)	3.0 (2.0, 6.0)	4.0 (3.0, 5.0)	1.0 (0.0, 3.0)
Min-Max	(1.0-21.0)	(1.0-19.0)	(1.0-11.0)	(0.0-21.0)
**Number of LBP Physical Therapy Visits within first 90 days**				
Mean (SD)	1.4 (1.5)	3.9 (3.2)	4.2 (3.2)	1.4 (2.5)
Median (Q1, Q3)	1.0 (1.0, 1.0)	3.0 (2.0, 5.0)	3.5 (2.0, 5.0)	1.0 (0.0, 1.0)
Min-Max	(1.0-9.0)	(1.0-17.0)	(1.0-11.0)	(0.0-17.0)

SD = standard deviation; Q = quartile

† - 88 participants (60.7%) in the Usual Care arm had physical therapy that started within 90 days from enrollment (N = 83 that had initiated physical therapy within 60 days from enrollment).

© 2023 Rhon DI et al. *JAMA Network Open.*

eTable 4. Summary of OSPRO-Yellow Flag Count by Treatment Group (Observed Values)

	Usual Care (N=145)	Risk Stratified (N=145)	Total (N=290)
**Baseline**			
Mean (SD)	5.7 (3.4)	5.3 (3.5)	5.5 (3.4)
Median (Q1, Q3)	6.0 (3.0, 9.0)	5.0 (2.0, 8.0)	6.0 (3.0, 8.0)
**Six-week**			
N	129	130	259
Mean (SD)	4.9 (3.6)	3.7 (3.3)	4.3 (3.5)
Median (Q1, Q3)	5.0 (1.0, 8.0)	2.0 (1.0, 6.0)	4.0 (1.0, 7.0)
**Six-month**			
N	116	115	231
Mean (SD)	5.1 (3.8)	4.3 (3.6)	4.7 (3.7)
Median (Q1, Q3)	4.5 (2.0, 9.0)	4.0 (1.0, 7.0)	4.0 (1.0, 8.0)
**One-year**			
N	112	121	233
Mean (SD)	5.2 (3.6)	4.8 (3.7)	4.9 (3.6)
Median (Q1, Q3)	5.0 (2.0, 8.0)	4.0 (1.0, 9.0)	4.0 (2.0, 8.0)

OSPRO-YF = Optimal Screening for Prediction of Referral and Outcome; SD = standard deviation; Q = quartile

eTable 5. Mean RMDQ Scores at Each Time Point by Treatment Group (Observed Values)

	Usual Care (N=145)	Risk Stratified Care (N=145)	Total (N=290)
**Baseline RMDQ Total Score (0-24)**			
N	145	145	290
Mean (SD)	10.2 (5.8)	9.3 (5.9)	9.8 (5.8)
Median (Q1, Q3)	11.0 (6.0, 14.0)	8.0 (5.0, 14.0)	9.0 (5.0, 14.0)
Min-Max	(0.0-23.0)	(0.0-21.0)	(0.0-23.0)
**6-Week RMDQ Total Score (0-24)**			
N	126	128	254
Mean (SD)	5.5 (5.6)	4.5 (5.5)	5.0 (5.6)
Median (Q1, Q3)	4.0 (1.0, 9.0)	2.0 (0.0, 6.5)	3.0 (0.0, 8.0)
Min-Max	(0.0-23.0)	(0.0-22.0)	(0.0-23.0)
**6-Month RMDQ Total Score (0-24)**			
N	113	115	228
Mean (SD)	5.7 (6.1)	4.7 (5.5)	5.2 (5.8)
Median (Q1, Q3)	4.0 (0.0, 10.0)	2.0 (0.0, 8.0)	3.0 (0.0, 8.0)
Min-Max	(0.0-24.0)	(0.0-21.0)	(0.0-24.0)
**1-Year RMDQ Total Score (0-24)**			
N	113	119	232
Mean (SD)	5.8 (5.9)	5.5 (6.4)	5.6 (6.2)
Median (Q1, Q3)	3.0 (1.0, 10.0)	3.0 (0.0, 8.0)	3.0 (0.0, 10.0)
Min-Max	(0.0-23.0)	(0.0-24.0)	(0.0-24.0)

SD = standard deviation; Q = quartile RMDQ = Roland Morris Disability Questionnaire

© 2023 Rhon DI et al. *JAMA Network Open.*

eTable 6. Mean PROMIS Pain Interference Scores at Each Time Point by Treatment Group (Observed Values)

	Usual Care (N=145)	Risk Stratified Care (N=145)	Total (N=290)
**Baseline**			
N	145	145	290
Mean (SD)	58.0 (6.8)	56.5 (7.5)	57.2 (7.2)
Median (Q1, Q3)	57.6 (53.7, 61.6)	56.6 (52.4, 62.3)	57.0 (53.3, 62.1)
Min-Max	(40.7-77.0)	(40.7-77.0)	(40.7-77.0)
**6-Week**			
N	128	129	257
Mean (SD)	52.4 (7.5)	50.6 (7.5)	51.5 (7.5)
Median (Q1, Q3)	53.8 (47.8, 57.3)	51.4 (40.7, 55.9)	52.5 (40.7, 56.2)
Min-Max	(40.7-67.3)	(40.7-68.5)	(40.7-68.5)
**6-Month**			
N	113	115	228
Mean (SD)	52.2 (8.6)	50.4 (8.7)	51.3 (8.7)
Median (Q1, Q3)	53.3 (40.7, 57.9)	50.3 (40.7, 55.9)	52.3 (40.7, 57.1)
Min-Max	(40.7-77.0)	(40.7-77.0)	(40.7-77.0)
**1-Year**			
N	109	119	228
Mean (SD)	52.0 (8.9)	52.0 (8.1)	52.0 (8.5)
Median (Q1, Q3)	53.3 (40.7, 60.3)	52.5 (40.7, 57.2)	52.9 (40.7, 57.8)
Min-Max	(40.7-77.0)	(40.7-73.7)	(40.7-77.0)

PROMIS value T-scores are based on a standard score with mean of 50 and standard deviation of 10 in the reference population. SD = standard deviation; Q = quartile

© 2023 Rhon DI et al. *JAMA Network Open.*

eTable 7. Mean PROMIS Physical Function Scores at Each Time Point by Treatment Group (Observed Values)

	Usual Care (N=145)	Risk Stratified Care (N=145)	Total (N=290)
**Baseline**			
N	145	145	290
Mean (SD)	41.2 (6.3)	41.7 (6.6)	41.4 (6.4)
Median (Q1, Q3)	40.7 (37.6, 44.1)	40.8 (37.1, 44.9)	40.8 (37.3, 44.5)
Min-Max	(20.9-59.7)	(29.4-59.7)	(20.9-59.7)
**6-Week**			
N	128	129	257
Mean (SD)	45.7 (7.6)	46.3 (7.1)	46.0 (7.4)
Median (Q1, Q3)	43.4 (40.8, 49.1)	45.2 (41.3, 50.1)	44.3 (41.1, 49.9)
Min-Max	(30.6-59.7)	(28.2-59.7)	(28.2-59.7)
**6-Month**			
N	113	115	228
Mean (SD)	45.2 (8.0)	47.0 (7.8)	46.1 (7.9)
Median (Q1, Q3)	43.4 (39.6, 48.5)	46.5 (41.3, 50.1)	44.4 (40.6, 50.1)
Min-Max	(26.9-59.7)	(28.3-59.7)	(26.9-59.7)
**1-Year**			
N	109	119	228
Mean (SD)	46.3 (8.1)	46.4 (7.4)	46.3 (7.7)
Median (Q1, Q3)	44.3 (40.2, 50.1)	45.2 (40.9, 50.1)	44.6 (40.7, 50.1)
Min-Max	(33.1-59.7)	(33.8-59.7)	(33.1-59.7)

PROMIS value T-scores are based on a standard score with mean of 50 and standard deviation of 10 in the reference population. SD = standard deviation; Q = quartile

© 2023 Rhon DI et al. *JAMA Network Open.*

eTable 8. Mean PROMIS Sleep Disturbance Scores at Each Time Point by Treatment Group (Observed Values)

	Usual Care (N=145)	Risk Stratified Care (N=145)	Total (N=290)
**Baseline**			
N	145	145	290
Mean (SD)	53.2 (8.2)	53.6 (8.5)	53.4 (8.3)
Median (Q1, Q3)	53.9 (47.7, 58.2)	52.4 (48.6, 59.8)	53.1 (48.1, 58.9)
Min-Max	(30.5-77.5)	(30.5-77.5)	(30.5-77.5)
**6-Week**			
N	128	129	257
Mean (SD)	53.0 (9.5)	52.9 (8.6)	52.9 (9.1)
Median (Q1, Q3)	53.1 (46.6, 60.1)	53.5 (47.7, 58.0)	53.3 (47.0, 59.0)
Min-Max	(30.5-77.5)	(30.5-77.5)	(30.5-77.5)
**6-Month**			
N	113	115	228
Mean (SD)	52.8 (8.6)	51.8 (9.3)	52.3 (8.9)
Median (Q1, Q3)	52.7 (47.4, 59.3)	51.9 (46.0, 57.0)	52.5 (47.0, 57.7)
Min-Max	(30.5-77.5)	(30.5-77.5)	(30.5-77.5)
**1-Year**			
N	109	119	228
Mean (SD)	52.7 (9.6)	52.7 (8.7)	52.7 (9.1)
Median (Q1, Q3)	52.3 (45.6, 60.2)	53.1 (48.3, 58.5)	52.8 (47.0, 59.0)
Min-Max	(30.5-77.5)	(30.5-72.9)	(30.5-77.5)

PROMIS value T-scores are based on a standard score with mean of 50 and standard deviation of 10 in the reference population. SD = standard deviation; Q = quartile

© 2023 Rhon DI et al. *JAMA Network Open.*

eTable 9. COVID-19 Impact by Treatment Group

Question<br>**How has the coronavirus pandemic affected your...?**	6 Months<br>N=10		1 Year<br>N=39	
	Risk-Stratified Care (n=6)	Usual Care (n=4)	Risk-Stratified Care (n=23)	Usual Care (n=16)
Ability to get healthcare for your back pain (including pain treatment, prescription and over-the-counter medications, medical and mental health visits, other treatments)				
Reduced my ability to get healthcare A LOT.	2 (33.3%)	2 (50.0%)	4 (17.4%)	3 (18.8%)
Reduced my ability to get healthcare A LITTLE	0 (0.0%)	0 (0.0%)	7 (30.4%)	7 (43.8%)
NOT AFFECTED my ability to get healthcare.	3 (50.0%)	3 (75.0%)	12 (52.2%)	6 (37.5%)
IMPROVED my ability to get healthcare.	0 (0.0%)	0 (0.0%)	0 (0.0%)	0 (0.0%)
Level of back pain (including frequency and duration)				
My back pain is A LOT WORSE	2 (33.3%)	1 (25.0%)	3 (13.0%)	1 (6.25%)
My back pain is A LITTLE WORSE	1 (16.7%)	1 (25.0%)	6 (26.1%)	4 (25.0%)
NOT AFFECTED my back pain	3 (50.0%)	2 (50.0%)	12 (52.2%)	10 (62.5%)
IMPROVED my back pain	0 (0.0%)	0 (0.0%)	2 (8.7%)	1 (6.25%)
Ability to continue treatment goals using self-management or other treatment strategies (including exercise, physical activity, therapy, etc.)				
I have made NO progress on treatment goals.	2 (3.33%)	1 (25.0%)	6 (26.1%)	3 (18.8%)
I have made LITTLE progress on treatment goals.	1 (16.7%)	2 50.0%)	4 (17.4%)	10 (62.5%)
I have made SOME progress on treatment goals.	2 (33.3%)	1 (25.0%)	10 (43.5%)	3 (18.8%)
I have made SIGNIFICANT progress on my treatment goals.	1 (16.7%)	0 (0.0%)	3 (13.0%)	0 (0.0%)

© 2023 Rhon DI et al. *JAMA Network Open.*

eTable 10. Usual Care Intervention Recommended Reporting Elements

Recommend Item[1]	Description of Care
**1. Type of Practitioner Delivering Care**	Primary Care Providers (Clinicians in Family Medicine/Primary Care: Physicians, Physicians Assistants, Nurse Practitioners) screened and referred patients to the study. Any referrals to physical therapy (PT) were placed by the PCP outside the study, and then PT would have been delivered by Physical Therapists who did not receive any of the risk-stratified care training.
**2. Self-Management Education**	All patients were provided a copy of the education pamphlet with guidance for patients based on the DoD-VA low back pain guidelines[2] and watched the Truth About Low Back Pain educational video.[3] Primary Care Providers were asked to reinforce these messages with all of their patients.
**3. Physical Activity and Movement were addressed**	These components of self-management were addressed to the extend present in the educational pamphlet; and Primary Care Providers were reminded to reinforce these key messages (but fidelity or quality were not assessed; nor extent to which additional education or variations in educational delivery existed between different clinicians). Guidance to remain active even if the pain has not gone away and guidance for activity were provided in the Truth About Low Back Pain educational video and also present in the DoD-VA low back pain guidelines patient handout.[4]
**4. Type of medication used**	Patients were not stopped from taking any medication prescribed by their PCP. PCPs were asked to consider minimizing any additional care (i.e., pain medication, imaging, referrals) for anyone potentially eligible, but none of these were strictly prohibited. The self-management education from the DoD-VA low back pain guidelines provides information about the efficacy and long-term effectiveness of various pain medications. Actual pain medication received during the first 90 days including initial consultation as well as pain medications for the entire 1-year follow-up are listed in manuscript Table 5.
**5. Dose of the intervention (including frequency/duration)**	The amount of care varied based on what the primary care provider felt was a most adequate treatment for each patient. Some individuals may have been referred to PT as part of the usual care pathway but would not have seen a study-trained Physical Therapist. Use of PT (number of visits) across both intervention groups, both within the first 90 days and over the full 1-year follow-up are listed in the manuscript Table 5, as well as medications, referrals, and imaging studies.
**6 . Consistent with current guidelines**	The current DoD-VA low back pain guidelines provide 24 non-invasive and non-surgical interventions that carry a recommendation of neutral or "recommended for".[5] The earlier guidelines present when this trial began had 9 recommended interventions.[6] All participants received the patient education pamphlet developed by the DoD-VA low back pain working group which summarized the guideline recommendations.[4] The extent to which all subsequent care actually aligned with these recommendations is unknown and was not tracked. However, common healthcare utilization elements received by all patients are present in manuscript Table 5.

Note: Usual Care elements are described according to recommendations for describing usual care treatment arms for low back pain care by Pascoe et al.[1]

© 2023 Rhon DI et al. *JAMA Network Open.*

eReferences

1 Pascoe SC, Spoonemore SL Jr, Young JL, Rhon DI. Proposing six criteria to improve reproducibility of 'usual care' interventions in back pain trials: a systematic review. *J Clin Epidemiol* 2022; **149**: 227–35.

2 Pangarkar SS, Kang DG, Sandbrink F, *et al.* VA/DoD Clinical Practice Guideline: Diagnosis and Treatment of Low Back Pain. *J Gen Intern Med* 2019; **34**: 2620–9.

3 Clinically Relevant Technologies. The Truth About Low Back Pain. App Store. 2018; published online March 14. https://apps.apple.com/us/app/the-truth-about-low-back-pain/id1325643930 (accessed March 12, 2020).

4 VA/DoD Clinical Practice Guideline for Diagnosis and Treatment of Low Back Pain. Managing Low Back Pain patient education booklet. 2017. https://www.qmo.amedd.army.mil/lbp/Final_LBP_Booklet.pdf.

5 VA/DoD Clinical Practice Guidelines - Diagnosis and Treatment of Low Back Pain - Provider Summary. Department of Veterans Affairs; Department of Defense, 2022 https://www.healthquality.va.gov/guidelines/Pain/lbp/VADoDLBPCPGProviderSummaryFinal508.pdf.

6 VA/DOD Cinical Practice Guideline for Diagnosis and Treatment of Low Back Pain Guideline Summary. Veterans Affairs Dept., Office of Quality, Safety, and Value, 2018.

© 2023 Rhon DI et al. *JAMA Network Open.*
