# Supplementary material for: Effect of Risk-Stratified Care on Disability Among Adults With Low Back Pain Treated in the Military Health System: A Randomized Clinical Trial
Source: JAMA Netw Open. 2023 Jul 6;6(7):e2321929. doi: 10.1001/jamanetworkopen.2023.21929 (PMC10326636; doi:10.1001/jamanetworkopen.2023.21929)
Supplement: Supplement 3. — Data Sharing Statement [file jamanetwopen-e2321929-s003.pdf]

Data Sharing Statement

Rhon. Effect of Risk-Stratified Care on Disability Among Adults With Low Back Pain Being Treated in the Military Health System. *JAMA Netw Open*. Published July 06, 2023. doi:10.1001/jamanetworkopen.2023.21929

Data
Data available: Yes
Data types: Deidentified participant data, Data dictionary
How to access data: Data will be shared upon reasonable request and after applicable Data Sharing Agreements are approved through the Defense Health Agency (found at www.health.mil)
When available: With publication

Supporting Documents
Document types: None

Additional Information
Who can access the data: Data will be shared upon reasonable request and after applicable Data Sharing Agreements are approved through the Defense Health Agency (found at www.health.mil)
Types of analyses: For the purposes specified when submitting a reasonable request.
Mechanisms of data availability: Data will be made available with investigator support, proper plan for the data, and after applicable Data Sharing Agreements are approved through the Defense Health Agency (found at www.health.mil)
